# Indexed Aortic Valve Calcium Volume by Computed Tomography Angiography in Patients With Aortic Stenosis: Results of an International Multicenter Cohort Study

**DOI:** 10.1016/j.jcmg.2025.09.013

**Published:** 2025-10-25

**Authors:** Jolien Geers, Neil Craig, Kajetan Grodecki, Maria Lembo, Shruti S. Joshi, Trisha Singh, Rong Bing, Jacek Kwieciński, Lorenzo Carnevale, Dorien Kimenai, Soongu Kwak, Seung-Pyo Lee, Kush Patel, Thomas Treibel, Aroa Ruiz Muñoz, Jose F. Rodriguez Palomares, Jae-Kwan Song, Marie-Annick Clavel, Pamela Piña, Daniel Lorenzatti, Azeem Latib, Leandro Slipczuk, Ronak Rajani, Vitaliy Androshchuk, Julia Niemierko, Marek Grygier, Andrzej Walczak, Dariusz Jagielak, Marcin Protasiewicz, Wojciech Wojakowski, Janusz Kochman, Zenon Huczek, Nils Sofus Borg Mogensen, Jordi Sanchez Dahl, Vivek Patel, Tulika Garg, Hasanian Al-Jilaihawi, Raj Makkar, Steven Droogmans, Bernard Cosyns, Piotr Slomka, Michelle C. Williams, David E. Newby, Damini Dey, Marc R. Dweck

**Affiliations:** a BHF Centre for Cardiovascular Science, University of Edinburgh, Edinburgh, United Kingdom;; b Departments of Biomedical Sciences and Medicine, Cedars-Sinai Medical Center, Biomedical Imaging Research Institute, Los Angeles, California, USA;; c Department of Cardiology, Universitair Ziekenhuis Brussel (UZ Brussel), Vrije Universiteit Brussel (VUB), Brussels, Belgium;; d 1st Department of Cardiology, Medical University of Warsaw, Warsaw, Poland;; e Department of Advanced Biomedical Sciences, Federico II University of Naples, Naples, Italy;; f Department of Interventional Cardiology and Angiology, Institute of Cardiology, Warsaw, Poland;; g Department of AngioCardioNeurology and Translational Medicine, IRCCS Neuromed, Pozzilli, Italy;; h Department of Internal Medicine, Seoul National University Hospital, Seoul, South Korea;; i Barts Health NHS Trust and University College London, London, United Kingdom;; j Department of Cardiology, Vall d’Hebron Hospital Universitari, Vall d’Hebron Barcelona Hospital Campus, Barcelona, Spain;; k Cardiovascular Diseases, Vall d’Hebron Institut de Recerca (VHIR), Vall d’Hebron Barcelona Hospital Campus, Barcelona, Spain;; l Department of Medicine, Universitat Autònoma de Barcelona, Bellaterra, Spain;; m CIBER de Enfermedades Cardiovasculares, Instituto de Salud Carlos III, Madrid, Spain;; n Division of Cardiology, Asan Medical Center Heart Institute, University of Ulsan College of Medicine, Seoul, Republic of Korea;; o Institut Universitaire de Cardiologie et de Pneumologie de Québec/Québec Heart and Lung Institute, Université Laval, Québec City, Québec, Canada;; p Cardiology Division, Montefiore Healthcare Network/Albert Einstein College of Medicine, New York, New York, USA;; q Department of Cardiology and Cardiac Surgery, Guy’s and St Thomas’ Hospitals NHS Foundation Trust, London, United Kingdom;; r 2nd Department of Radiology, Medical University of Gdansk, Gdansk, Poland;; s 12th Department of Cardiology, Poznan University of Medical Sciences, Poznan, Poland;; t Department of Cardiac Surgery, Medical University of Lodz, Lodz, Poland;; u Department of Cardiac Surgery, Medical University of Gdansk, Gdansk, Poland;; v Department of Cardiology, Wroclaw Medical University, Wroclaw, Poland;; w Division of Cardiology and Structural Heart Diseases, Medical University of Silesia, Katowice, Poland;; x Department of Cardiology, Odense University Hospital, Odense, Denmark;; y Cedars-Sinai Smidt Heart Institute, Cedars-Sinai Medical Center, Los Angeles, California, USA.

**Keywords:** aortic valve calcium score, aortic valve stenosis, computed tomography angiography, prognosis

## Abstract

**BACKGROUND:**

Calcium scoring from noncontrast computed tomography (CT) is used clinically to adjudicate aortic stenosis severity in patients with discordant echocardiography.

**OBJECTIVES:**

The aim of this study was to investigate whether quantification of aortic valve calcium volume from computed tomography angiography (CTA) can provide robust diagnostic discrimination of disease severity and inform risk stratification of patients with aortic stenosis.

**METHODS:**

Patients with mild to severe aortic stenosis who underwent concurrent CTA and echocardiography were included in a retrospective international multicenter observational cohort study. Accuracy of aortic valve calcium volume to diagnose severe aortic stenosis in patients with concordant disease on echocardiography was assessed. Association of aortic valve calcium volume with the incidence of aortic valve replacement or all-cause death was investigated.

**RESULTS:**

The study included 1,521 patients (mean age: 74 ± 10 years; 44% female; median peak aortic jet velocity: 3.8 m/s [Q1-Q3: 3.1–4.5 m/s]). Indexed aortic valve calcium volume correlated with peak aortic jet velocity (ρ = 0.723; *P* < 0.001) and noncontrast CT calcium score (ρ = 0.896; *P* < 0.001). In the derivation cohort (n = 689), sex-specific thresholds for indexed calcium volume (men: 122 mm^3^/cm^2^; women: 61 mm^3^/cm^2^) provided excellent diagnostic discrimination for severe aortic stenosis (C-statistic: 0.900 for men; 0.926 for women). Similar diagnostic discrimination was observed in the validation cohort (n = 459; C-statistic: 0.933 for men; 0.944 for women). Clinical outcomes were available in 711 patients (25% with discordant echocardiography), with 249 reaching the primary endpoint after 26 months (Q1-Q3: 12–53 months). Indexed calcium volume thresholds were independently associated with aortic valve replacement or all-cause mortality in both the cohort as a whole (HR: 2.01 [95% CI: 1.30–3.10]; *P* < 0.01) and those with discordant echocardiography (HR: 1.58 [95% CI: 1.01–2.44]).

**CONCLUSIONS:**

In patients with aortic stenosis, indexed aortic valve calcium volume from CTA provides accurate discrimination of disease severity and additive prognostic information. This technique can be easily applied to patients undergoing CTA for transcatheter aortic valve replacement or coronary artery evaluation without the need for a separate noncontrast CT scan.

## CENTRAL ILLUSTRATION

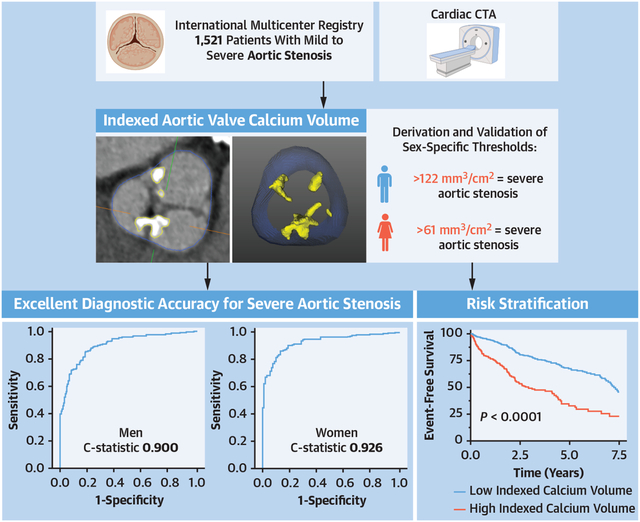

Indexed Aortic Valve Calcium Volume Thresholds for Diagnosis and Prognosis of Patients With Aortic Stenosis

In an international multicenter registry of >1,500 patients with all degrees of aortic stenosis severity undergoing CTA, we derived and validated sex-specific thresholds of indexed aortic valve calcium volume, which are 122 mm^3^/cm^2^ for men and 61 mm^3^/cm^2^ for women. These thresholds demonstrate excellent diagnostic accuracy for severe aortic stenosis and provide powerful prognostic information, namely predict risk of aortic valve replacement or death in patients with aortic stenosis. Receiver-operator characteristic curve analysis represents results of the derivation cohort (n = 689). Kaplan-Meier curve represents results of patients who had not already been referred for valve intervention and who had outcome data available (n = 711). CT = computed tomography; CTA = computed tomography angiography.

Aortic stenosis is a prevalent potentially life-threatening condition in which progressive fibrocalcific changes within the valve cusps lead to valve stiffening and obstruction.^1–3^ It remains one of the last major cardiovascular conditions without effective disease-modifying medical therapy, and timely referral of patients with severe symptomatic stenosis for aortic valve replacement is essential to prevent adverse clinical events.^4^ Echocardiography is the principal imaging modality for assessment of aortic stenosis severity but has several limitations, with dynamic measures of stenosis severity dependent on hemodynamic state and transaortic flow, leading to discordant measurements in up to one-third of patients.^5^ This leads to diagnostic uncertainty, delays in management, and potentially adverse clinical outcomes.

Noncontrast computed tomography (CT) aortic valve calcium scoring is an established adjunct to echocardiography when measures of disease severity are discordant. It provides an anatomic, flow-independent, quantitative measure of valvular calcium which has been validated against echocardiography and is well suited for prediction and monitoring of disease progression.^5–7^ However, the unenhanced scan technique provides limited anatomic information about valve morphology and exact distribution of calcium, which can lead to potential measurement errors. There is increasing interest in quantifying aortic valve calcium from computed tomography angiography (CTA), the reference imaging modality for transcatheter aortic valve replacement^8^ and widely used for the assessment of coronary artery disease.^9^ This approach would allow for more accurate anatomic assessment, and would obviate the need for a dedicated noncontrast CT scan in patients already undergoing CTA. The present study aimed to investigate the clinical utility of aortic valve calcium volume quantified by CTA in an international multicenter registry of patients with aortic stenosis. Specifically, we sought to establish and validate indexed aortic valve calcium volume thresholds and to determine their ability to arbitrate disease severity and to inform the occurrence of future clinical events.

## METHODS

### STUDY DESIGN AND STUDY POPULATION.

This is a retrospective multicenter observational cohort study. International centers (Supplemental Table 1) contributed clinical, echocardiographic, and CT data from patients with at least mild nonrheumatic valvular aortic stenosis (defined as peak aortic jet velocity ≥2.0 m/s and mean gradient <20 mm Hg^10^) into a multicenter registry. Patients with a history of aortic valve replacement were excluded. Patients were required to have undergone echocardiography and contrast-enhanced cardiac CT within 3 months between 2002 and 2023. Centers contributed data from prospective clinical research studies, or from patients undergoing cardiac CT for clinical indications. Clinical indications to perform cardiac CT included evaluation of coronary artery disease or aortic stenosis, and preoperative evaluation before aortic valve replacement. Informed consent (including data sharing) and institutional review board approval were obtained for patients who had taken part in research studies. All CT scans were deidentified before transfer to the core laboratory (University of Edinburgh). Data analysis was performed between January and August 2024. The study was conducted in accordance with the Declaration of Helsinki. This report adheres to the STROBE (Strengthening the Reporting of Observational Studies in Epidemiology) reporting guidelines (Supplemental Table 2).

### CLINICAL AND IMAGING DATA.

An online data collection form with requisite clinical information was completed by each participating site and included age, sex, cardiovascular risk factors, medical history, and outcome data where available.

#### Echocardiography.

Echocardiography was performed locally according to international guidelines^11–13^ and included peak aortic jet velocity, mean gradient, aortic valve area (continuity equation), left ventricular ejection fraction, and indexed stroke volume. On the basis of these measures, patients were categorized as follows:^10,14^ 1) concordant severe aortic stenosis; 2) concordant nonsevere aortic stenosis; 3) discordant low-flow, low-gradient aortic stenosis with reduced ejection fraction; 4) discordant low-flow, low-gradient aortic stenosis with preserved ejection fraction; 5) discordant normal-flow, low-gradient aortic stenosis; and 6) discordant normal-flow, high-gradient aortic stenosis (Supplemental Figure 1).

#### Noncontrast CT aortic valve calcium scoring.

**FIGURE 1:**
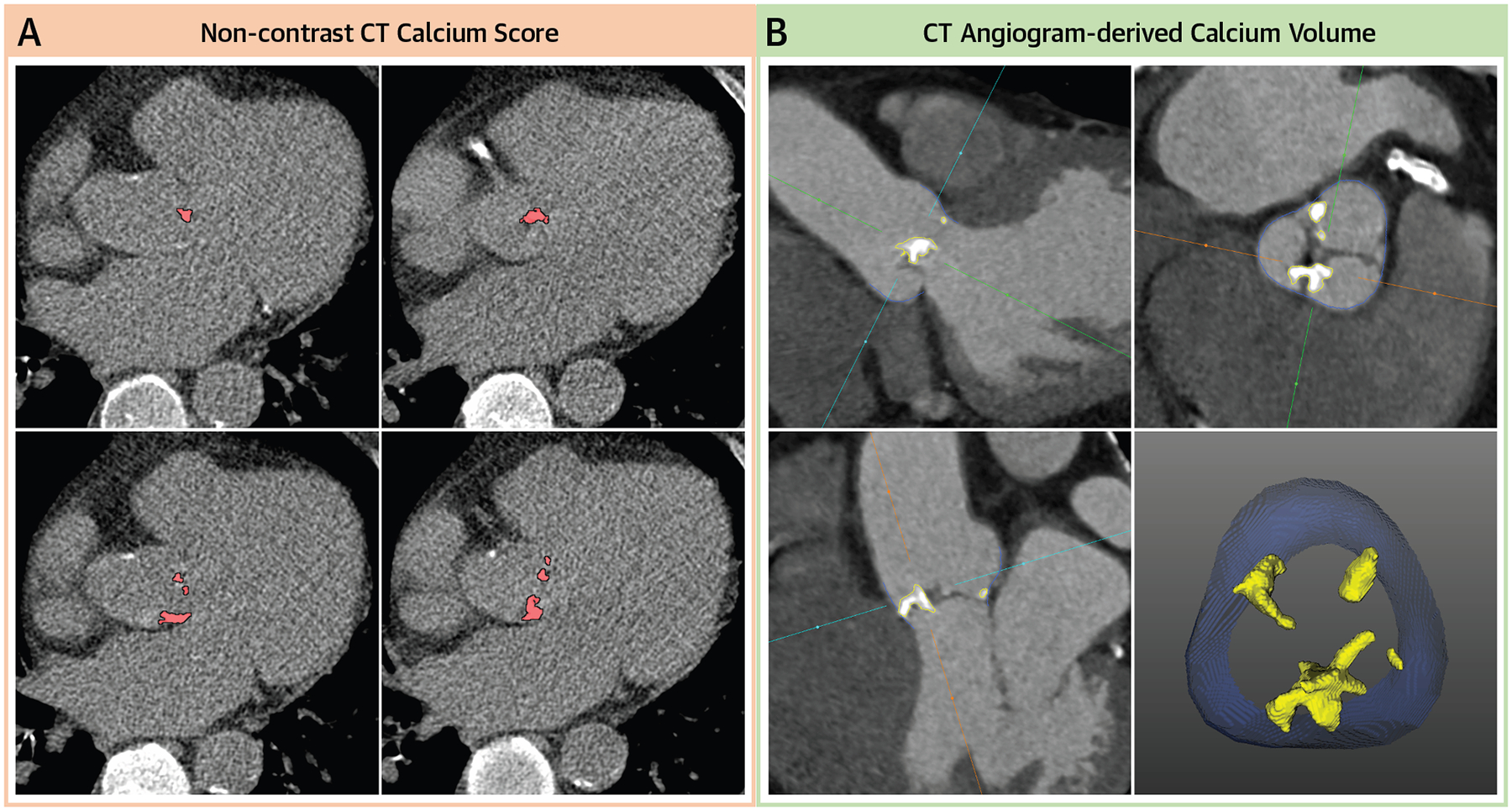
Quantification of Aortic Valve Calcification by CT Aortic valve calcium quantified by (A) noncontrast CT calcium score and (B) CT angiogram–derived calcium volume in a male patient with moderate aortic stenosis on echocardiography, with a calcium score of 1,620 AU and calcium volume indexed to annulus area of 110 mm^3^/cm^2^; both modalities confirm nonsevere disease. AU = Agatston Units; CT = computed tomography.

Noncontrast CT scans were performed at the discretion of the attending clinician and available in a subgroup of the total study population. All scans were gated from 70% to 80% of the R-R interval, with a tube current of 40 to 1,350 mA and a voltage of 120 kV. Image data were reconstructed using a slice thickness of 3 mm. The aortic valve calcium score was quantified as described previously^15^ (Figure 1A). Severe calcification was defined as a calcium score >1,200 Agatston Units (AU) in women and >2,000 AU in men.^10,14^

### CTA.

All centers performed electrocardiographygated contrast-enhanced CT angiograms acquired in diastole with the aortic valve in a closed position using tube current of 250–850 mA and a voltage of 100–120 kV. Image data were reconstructed with a slice thickness of 0.6–0.75 mm. Imaging was performed on a range of different scanners (Supplemental Table 1).

### CALCIUM VOLUME ANALYSIS.

Aortic valve calcium volume analysis was performed in the core laboratory by 2 experienced readers (J.G., K.G.) blinded to the clinical, echocardiographic, and outcome data. The computation of aortic valve calcium volume was performed using a diastolic phase (70% R-R interval) of contrast-enhanced CT using a semiautomated software (ValveQuant module, Autoplaque version 2.5; Cedars-Sinai Medical Center) for scan-specific thresholding and identification of calcific tissue within the aortic valve region of interest using an integrated Gaussian mixture model as described previously.^16–18^ The analysis (~5 minutes per scan) includes the following steps: 1) orientation of the CT scan into the annular plane;^19^ 2) incorporation of the entire aortic valve from the annular plane to origin of the right coronary artery; 3) automated division of the volume of interest into 5 slices, allowing manual adjustment to exclude calcific tissue arising from nonvalvular structures; and 4) automated identification and calculation of all tissue above the calcium threshold (based on the integrated Gaussian mixture model, setting a threshold of 99.7 percentile of blood pool CT attenuation) (Figure 1B). To account for differences in valve size, the calcium volume was indexed to the annulus area (indexed aortic valve calcium volume).

### REPEATABILITY.

Intraobserver repeatability of indexed aortic valve calcium volume was assessed on 50 random and anonymized CT scans by the same observer (J.G.) on 2 occasions at least 4 weeks apart in random order to minimize recall bias. To evaluate interobserver repeatability, 50 scans were independently analyzed by 2 trained observers (J.G., K.G.) 4 weeks apart.

### CLINICAL OUTCOMES.

The primary endpoint was defined as the time to first event of aortic valve replacement (transcatheter or surgical) or all-cause death. Decisions regarding valve intervention were made by the local multidisciplinary team according to international clinical guidelines,^10,14^ blinded to the result of the indexed aortic valve calcium volume. Patients in whom a decision to proceed to valve replacement was already made at time of CT imaging were excluded from the outcome analysis.

### STATISTICAL ANALYSIS.

Continuous variables are presented as mean ± SD or median (Q1-Q3) as appropriate. Categorical variables are expressed as counts and percentages. Comparison of continuous variables was performed using an independent samples Student’s *t*-test or Mann-Whitney U test as appropriate; categorical variables were evaluated using the chi-square test. Intraobserver and interobserver repeatability were assessed using intraclass correlation coefficient (2-way agreement model) and Bland-Altman analysis. Correlations between continuous variables were assessed with linear regression analysis and either Pearson *r* or Spearman ρ, and interpreted as follows: correlation coefficients of <0.20 were categorized as very weak, 0.20 to <0.40 were categorized as weak, 0.40 to <0.60 were categorized as moderate, 0.60 to <0.80 were categorized as strong, and 0.80 to 1.00 were categorized as very strong.

For derivation and validation of optimal thresholds to diagnose severe aortic stenosis, all patients with concordant echocardiographic grading from each center were split into 2 groups based on scan dates (Supplemental Figure 2). The earlier scan dates were included in the derivation cohort (60%), and the later in the validation cohort (40%). Receiver-operating characteristic curve analysis was used to determine optimal indexed aortic valve calcium volume thresholds with sensitivity and specificity to identify severe aortic stenosis. Discriminatory performance of different parameters was compared using the DeLong method.

Kaplan-Meier curves and log-rank tests of the time to the primary endpoint were used to compare survival according to indexed aortic valve calcium volume. Univariable and multivariable Cox proportional hazards regression analyses were used to determine associations between the indexed aortic valve calcium volume, clinical characteristics, and the primary endpoint. The proportional hazards assumption was tested by plotting Schoenfeld residuals. The multivariable Cox proportional hazards model was adjusted for variables significantly associated (ie; *P* < 0.05) with the primary endpoint on univariate analysis, and known risk factors. To achieve normal distribution, we applied log_2_ transformation for aortic jet velocity. To assess potential differences between sex and the indexed aortic valve calcium volume, an interaction term was included in the multivariable Cox model. A 2-sided value of *P* < 0.05 was considered statistically significant. Statistical analyses were performed using SPSS (version 29.0.1.0, IBM) and R studio (version 2023.12.0, Posit).

## RESULTS

### STUDY POPULATION.

A total of 1,672 patients were recruited from 14 international centers; of these, 151 (9%) were excluded due to inadequate image quality (Supplemental Figure 2). The study population (N = 1,521) had a mean age of 74 ± 10 years; 44% were female. The median aortic peak velocity was 3.8 m/s (Q1-Q3: 3.1–4.5 m/s), and the median valve area was 0.83 cm^2^ (Q1-Q3: 0.66–1.14 cm^2^). Concordant echocardiographic grading was present in 1,148 (76%) patients, of whom 661 (58%) had severe disease (Table 1).

In multivariable analysis, male (β = 51.2 [95% CI: 36.1–66.3]; *P* < 0.001), older age (β = 3.0 [95% CI: 2.3–3.8]; *P* < 0.001), coronary artery disease (β = 40.2 [95% CI: 22.6–57.8]; *P* < 0.001), bicuspid aortic valve (β = 96.4 [95% CI: 75.5–117.3]; *P* < 0.001), and left ventricular ejection fraction (β = −1.3 [95% CI: −2.1 to −0.5]; *P* = 0.003) were independently associated with indexed aortic valve calcium volume (Supplemental Table 3).

Sensitivity analysis showed differences in clinical characteristics and valve morphology between White and Asian patients, with White patients having more comorbidities and Asian patients having a higher prevalence of bicuspid aortic valve disease (Supplemental Table 4). However, ethnicity was not an independent predictor of aortic valve calcium volume (Supplemental Table 5), indicating that it does not directly influence calcification burden.

### REPEATABILITY OF INDEXED AORTIC VALVE CALCIUM VOLUME ANALYSIS.

The indexed aortic valve calcium volume demonstrated excellent intraobserver and interobserver repeatability with correlation coefficients of 0.998 and 0.996, respectively. There were tight limits of agreement and no fixed or proportional biases (Supplemental Table 6, Supplemental Figure 3).

### PATIENTS WITH CONCORDANT GRADING ON ECHOCARDIOGRAPHY.

In 1,148 patients with concordant aortic stenosis, the indexed aortic valve calcium volume was 110 mm^3^/cm^2^ (Q1-Q3: 38–225 mm^3^/cm^2^), and this was higher in those with severe disease (194 mm^3^/cm^2^ [Q1-Q3: 115–313 mm^3^/cm^2^] vs 37 mm^3^/cm^2^; [Q1-Q3: 13–75 mm^3^/cm^2^]; *P* < 0.001) and men (234 mm^3^/cm^2^ [Q1-Q3: 145–363 mm^3^/cm^2^] vs 154 mm^3^/cm^2^ [Q1-Q3: 93–254 mm^3^/cm^2^]; *P* < 0.001) (Supplemental Figure 4). It correlated strongly with echocardiographic measures of stenosis severity and very strongly with noncontrast CT calcium score (Supplemental Table 7, Supplemental Figure 5).

The derivation cohort consisted of 689 patients; of these, 60% had severe disease on echocardiography (Supplemental Table 8). The optimal indexed aortic valve calcium volume threshold for severe aortic stenosis was 122 mm^3^/cm^2^ in men and 61 mm^3^/cm^2^ in women (Supplemental Table 9), providing excellent diagnostic discrimination for severe aortic stenosis (C-statistic: 0.900 in men; 0.926 in women). A total of 387 patients also had noncontrast CT aortic valve calcium scores available, and the diagnostic discriminations were similar: C-statistic 0.887 for indexed calcium volume compared with 0.871 for calcium score in men, and 0.934 for indexed calcium volume compared with 0.922 for calcium score in women (Supplemental Table 9). Sensitivity analysis showed that nonindexed aortic valve calcium volume thresholds for severe aortic stenosis were 619 mm^3^ in men and 273 mm^3^ in women. Nonindexed calcium volume demonstrated worse diagnostic discrimination for severe aortic stenosis in men (C-statistic: 0.890; *P*_deLong_ = 0.014) than the indexed calcium volumes but performed comparably in women (C-statistic: 0.922; *P*_deLong_ = 0.250). Based on this finding, outcome analyses were conducted using indexed calcium volume only (results of nonindexed volumes can be found in the Supplemental Tables 10 and 11).

The validation cohort included 459 patients with 246 (54%) having severe disease (Supplemental Table 12). The optimal thresholds from the derivation cohort performed equally well with a sensitivity of 83% and specificity of 87% in men (C-statistic: 0.933), and a sensitivity of 87% and specificity of 91% in women (C-statistic: 0.944) (Supplemental Table 9). The optimal indexed aortic valve calcium volume thresholds determined in the validation cohort were identical in men (122 mm^3^/cm^2^) and nearly identical in women (67 mm^3^/cm^2^). Sensitivity analysis showed that the optimal thresholds from the derivation cohort for nonindexed calcium volume performed similarly to indexed volumes in men (C-statistic: 0.926, *P*_deLong_ = 0.222) with a sensitivity of 77% and specificity of 86%, and in women (C-statistic: 0.935, *P*_deLong_ = 0.067) with sensitivity of 83% and specificity of 91%.

### CLINICAL OUTCOMES.

After exclusion of patients already referred for valve intervention (n = 629) and those with no available outcome data (n = 181), 711 (47%) patients were followed for a median of 26 months (Q1-Q3: 12–53 months). In total, 249 (35%) patients experienced the primary endpoint: 152 patients underwent aortic valve replacement and 97 died. These patients were older, were more likely to be male, had more severe aortic stenosis by echocardiography, and had higher indexed aortic valve calcium volume (Table 2).

**FIGURE 2:**
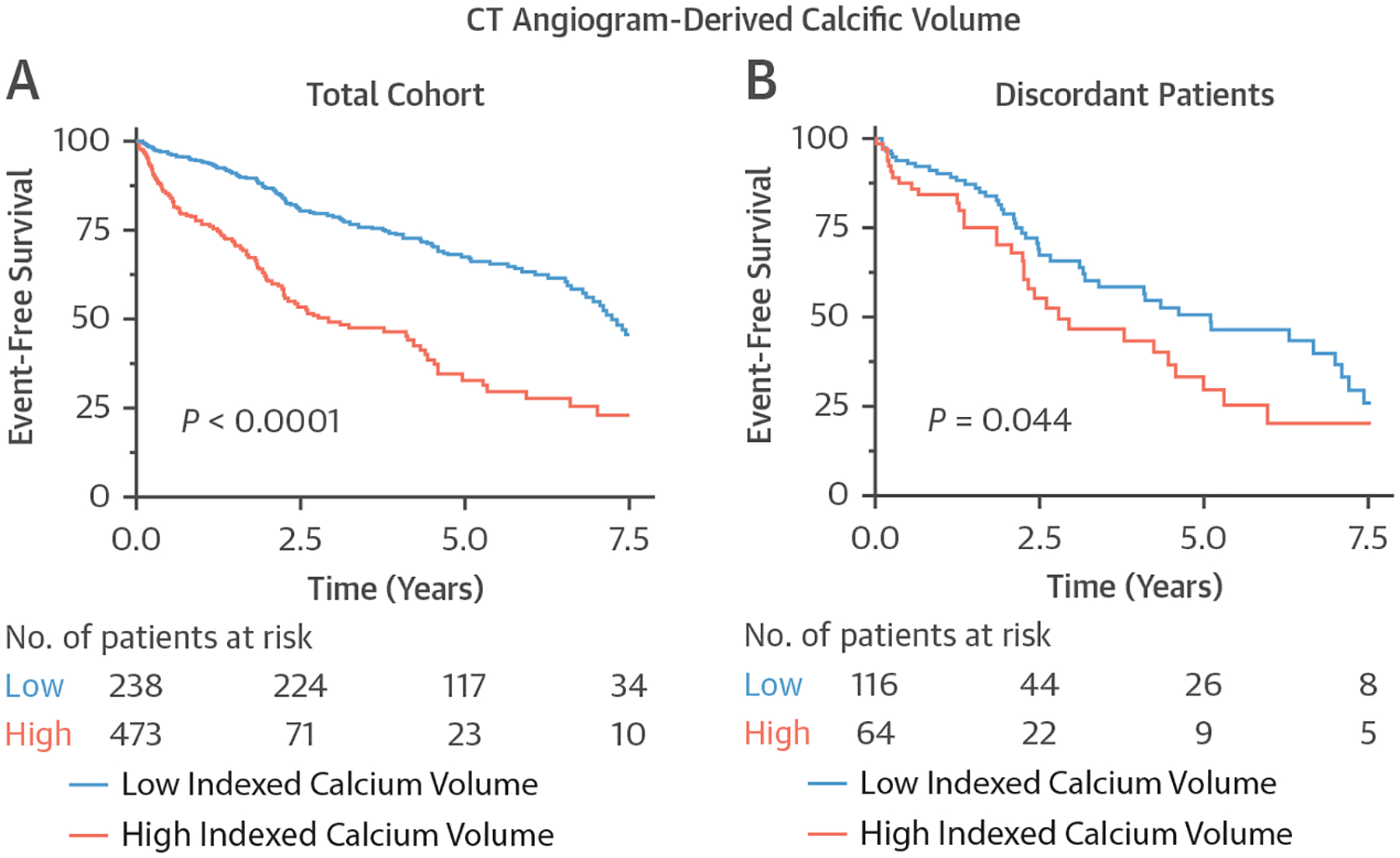
Association Between Aortic Valve Replacement or Death and Sex-Specific CT Angiogram–Derived Indexed Aortic Valve Calcium Volume Thresholds in Patients With Aortic Stenosis Kaplan-Meier curves demonstrating event-free survival using the sex-specific CT angiogram–derived indexed aortic valve calcium volume (61 mm^3^/cm^2^ in women and 122 mm^3^/cm^2^ in men) thresholds. Severe calcification was associated with adverse prognosis compared with nonsevere calcification, both in the total study cohort (log-rank *P* < 0.001) (A) and in a subgroup of patients with discordant echocardiographic measures of aortic stenosis disease severity (log-rank *P* = 0.044) (B). Abbreviation as in Figure 1.

On multivariable analysis, severe indexed aortic valve calcium volume was associated with an increased likelihood of aortic valve replacement or death (HR: 2.01 [95% CI: 1.30–3.10]; *P* < 0.01) (Figure 2, Supplemental Table 10). No interaction was observed between sex and the indexed aortic valve calcium volume on the primary endpoint (*P*_interaction_ = 0.22).

### PATIENTS WITH DISCORDANT GRADING ON ECHOCARDIOGRAPHY.

There were 373 patients (23%) with discordant echocardiographic disease severity findings: 217 with low-flow, low-gradient aortic stenosis (ejection fraction <50% [n = 77]; ejection fraction ≥50% [n = 140]), and 156 with normal-flow aortic stenosis (low gradient [n = 125]; high gradient [n = 31]) (Table 1). Indexed aortic valve calcium volume was lowest in patients with normal-flow, low-gradient aortic stenosis in both men and women (33% of men and 30% of women classified as severe aortic stenosis), and highest in male patients with normal-flow, high-gradient aortic stenosis (71% of patients having high indexed calcium volume) and in female patients with low-flow, low-gradient aortic stenosis with reduced ejection fraction (62% classified as severe aortic stenosis) (Supplemental Figure 6). Within the discordant group, male patients had higher calcium volume than female patients (Supplemental Figure 7).

After exclusion of patients already referred for valve intervention (n = 160) and those with no available data (n = 33), 84 of the 180 patients with follow-up data experienced the primary endpoint: 54 patients underwent aortic valve replacement, and 30 patients died. Severe indexed aortic valve calcium volume was associated with adverse clinical outcomes (log-rank *P* = 0.044). On multivariable analysis, severe indexed aortic valve calcium volume was associated with an increased likelihood of aortic valve replacement or death (HR: 1.58 [95% CI: 1.01–2.44]; *P* = 0.04) (Figure 2, Supplemental Table 11). There was no demonstrable interaction between sex, severe indexed aortic valve calcium volume, and the primary endpoint (*P*_interaction_ = 0.63).

## DISCUSSION

In this multicenter international registry, we have demonstrated that sex-specific indexed aortic valve calcium volume thresholds obtained from CTA provide excellent discrimination for diagnosing severe aortic stenosis and are independently associated with aortic valve replacement or death (Central Illustration). Given the routine use of CTA in clinical workflows, indexed aortic valve calcium volumes could be readily integrated into clinical practice as an alternative to calcium scoring, thereby eliminating the need for a separate dedicated noncontrast CT scan.

Up to one-third of patients with aortic stenosis have discordant echocardiographic measures,^5,20^ and international guidelines recommend noncontrast CT calcium scoring as a flow-independent arbitrator of severe aortic stenosis.^10,11,14^ However, patients with aortic stenosis often undergo contrast-enhanced CTA for evaluation of coronary artery disease or preprocedural planning before transcatheter aortic valve replacement, and the quantification of aortic valve calcium currently requires acquisition of a separate noncontrast CT scan introducing additional radiation exposure and logistical challenges. Obtaining all the necessary information from a single study, namely from CTA alone, is of great clinical interest because it provides superior anatomic information and avoids additional radiation exposure from an extra noncontrast CT scan. Our study presents a novel method to quantify aortic valve calcium from CTA with an integrated Gaussian mixture model. This model automatically derives a scan-specific, scanner-independent, and adaptive attenuation threshold for calcific tissue, which is based on the relative distribution of CT attenuation in the aortic valve and blood pool. This semiautomated approach enables calcium volume to be automatically and rapidly quantified with excellent intraobserver and interobserver repeatability. We have systematically applied this method to >1,500 patients with a broad range of aortic stenosis disease severity, recruited from multiple international centers across Europe, Asia, and North America, and evaluated on a range of scanners from different vendors. The obtained indexed aortic valve calcium volume demonstrated strong correlations with echocardiographic assessments and an excellent correlation with noncontrast CT calcium score. Moreover, our optimal sex-specific indexed aortic valve calcium volume thresholds were able to identify severe aortic stenosis with high sensitivity and specificity in both men and women. Our results were consistent across the derivation and validation cohort, demonstrating the robustness and generalizability of these indexed aortic valve calcium volumes thresholds in different patient populations of different ethnicities from around the world. We have therefore confirmed that indexed aortic valve calcium volume from contrast CT provides an alternative anatomic assessment of aortic stenosis severity to complement the hemodynamic information provided by echocardiography.

It is crucial to predict which patients with aortic stenosis are more likely to proceed to aortic valve replacement or who are at increased risk of death. Refining prognosis of patients with aortic stenosis can identify high-risk subgroups who might benefit from closer follow-up and timely intervention. Several studies have indicated that measuring aortic valve calcium from noncontrast CT provides important prognostic information beyond echocardiography.^6,7,21^ One of our key objectives was to assess the ability of our sex-specific indexed calcium volume thresholds to predict clinical outcomes. In a large subgroup of our population in whom prospective outcome data were available, we have demonstrated that severe indexed calcium volume can predict aortic valve replacement or all-cause death independent of clinical variables and standard echocardiographic assessments, thereby confirming the prognostic information of valvular calcium independent of the measurement technique.

One-quarter of our patients had discordant echocardiographic findings, and it is in this subpopulation that measuring aortic valve calcium could have the greatest benefit. In this cohort, indexed aortic valve calcium volume was able to stratify between patients with an increased risk of clinical events, was an independent predictor of aortic valve replacement or death on multivariable analysis, and again outperformed the prognostic ability of echocardiography assessment in this subpopulation. Our findings suggest that quantifying the aortic valve calcium burden by indexed calcium volume from CTA can provide valuable additional information to better characterize discordant patients and aid clinicians in decision-making.

The integration of indexed aortic valve calcium volume into clinical practice offers a practical alternative to noncontrast CT calcium scoring, providing comparable diagnostic and prognostic utility. This analysis can be quickly performed on standard cardiac CTA scans, which are routinely conducted in patients being evaluated for transcatheter aortic valve replacement, thus eliminating the need for an additional scan, reducing radiation exposure and streamlining workflow. Furthermore, contrast CTA allows for precise differentiation of valvular calcification from immediately adjacent calcification in the aortic root, left ventricular outflow tract, and mitral annulus, which can be challenging on axial noncontrast CT images. Additionally, it may be used opportunistically in patients undergoing CTA for coronary artery disease diagnosis, identifying those who may require echocardiographic assessment for aortic stenosis and those who would benefit from more frequent echocardiographic follow-up.

### STUDY LIMITATIONS.

Retrospective data collected between 2002 and 2023 were analyzed in this study, during which scanner technology, imaging techniques, and clinical practices may have changed. Certain patients included in the derivation cohort underwent CTA as work-up for valve intervention and thus were excluded from outcome analysis; however, we were able to assess clinical outcomes in many patients recruited as part of prospective cohort studies. Dobutamine stress echocardiography, which is recommended in discordant patients with low-flow states and left ventricular ejection fraction <50%,^10,14^ was not available. Similarly, echocardiographic measurements other than those used to define aortic stenosis severity were not uniformly available. Although calcium accumulation is likely to be the predominant factor contributing to stenosis severity in most patients, it is important to recognize that some patients exhibit atypically low calcium deposition.^22^ In these cases, noncalcific valve thickening might play a major role in the progression and severity of the disease. Although our calcium-focused approach demonstrated good diagnostic and prognostic abilities in the overall population, incorporation of noncalcific valve thickening could further enhance our understanding and improve patient-specific management strategies.^23^ Potential influence of confounding, mediation, or collinearity on the results in the subgroup with discordant echocardiographic findings cannot be excluded; thus, our findings require confirmation from larger prospective studies.

## CONCLUSIONS

In this large retrospective international multicenter registry of >1,500 patients with the full spectrum of aortic stenosis severity, we have developed and validated CT angiogram–derived sex-specific indexed aortic valve calcium volume thresholds. These demonstrate excellent diagnostic discrimination for severe disease and powerful prognostic information. This analysis technique can be performed quickly on standard cardiac CTA scans that are performed routinely in patients undergoing evaluation for transcatheter aortic valve replacement, eliminating the need for a separate noncontrast CT scan. Clinical translation of this novel anatomic assessment of aortic stenosis severity to aid in clinical decision for patients with discordant echocardiographic assessments is readily feasible and clinically robust.

## Supplementary Material

supplement

## Figures and Tables

**TABLE 1:** Characteristics of the Study Cohort

				Concordant		Discordant Low-Flow, Low-Gradient		Discordant Normal-Flow	
	All (N = 1,521)	Men (n = 851)	Women (n = 670)	Nonsevere (n = 487)	Severe (n = 661)	LVEF <50% (n = 77)	LVEF ≥50% (n = 140)	Low Gradient (n = 125)	High Gradient (n = 31)
Clinical									
Age, y	74 ± 10	73 ± 11	75 ± 10	71 ± 11	76 ± 10	78 ± 8	78 ± 9	74 ± 10	70 ± 12
Male	56	100	0	63	50	62	56	46	77
Body mass index, kg/m^2^	26 ± 5	26 ± 5	26 ± 5	26 ± 5	26 ± 5	27 ± 4	27 ± 5	25 ± 5	26 ± 5
Race									
Hispanic	0.6	0.5	0.7	1	0.3	0	0	0.8	3.2
White	54	55	52	39	58	91	82	39	39
Black	0.3	0.1	0.4	0.2	0.5	0	0	0	0
Asian	44	43	45	59	40	8	16	59	55
Unknown	1.7	1.5	1.9	1.8	1.8	1.3	1.4	0.8	3.2
Heart rate, beats/min	66 ± 13	64 ± 10	68 ± 14	65 ± 13	66 ± 12	75 ± 23	68 ± 17	65 ± 9	65 ± 11
Hypertension	71	71	71	70	70	78	81	66	71
Hyperlipidemia	52	51	53	45	56	69	66	34	42
Diabetes mellitus	29	30	27	27	30	39	29	27	19
Current or ex-smoker	31	43	12	31	24	58	49	30	43
Coronary artery disease	27	29	23	18	32	68	49	15	13
Bicuspid aortic valve	16	17	15	13	22	7	12	17	10
Echocardiography									
Peak velocity, m/s	3.8 (3.1–4.5)	3.8 (3.0–4.4)	4.0 (3.1–4.6)	2.8 (2.5–3.2)	4.6 (4.2–5.0)	3.2 (2.8–3.6)	3.5 (2.2–3.7)	3.6 (3.3–3.8)	4.2 (4.1–4.5)
Mean gradient, mm Hg	35 (21–48)	33 (20–46)	39 (23–50)	18 (13–24)	50 (43–61)	26 (20–33)	29 (24–35)	30 (24–35)	43 (39–47)
Aortic valve area, cm^2^	0.83 (0.66–1.14)	0.88 (0.70–1.20)	0.80 (0.62–1.03)	1.30 (1.14–1.55)	0.68 (0.55–0.79)	0.78 (0.65–0.85)	0.80 (0.65–0.87)	0.86 (0.78–0.92)	1.09 (1.01–1.22)
Aortic valve area index, cm^2^/m^2^	0.46 (0.36–0.64)	0.46 (0.37–0.64)	0.46 (0.36–0.64)	0.74 (0.63–0.89)	0.38 (0.31–0.44)	0.41 (0.32–0.45)	0.40 (0.35–0.46)	0.51 (0.45–0.56)	0.63 (0.52–0.71)
Stroke volume index, mL/m^2^	42 (35–50)	42 (35–49)	43 (35–52)	45 (38–52)	43 (36–50)	25 (22–30)	29 (25–32)	42 (38–48)	55 (46–67)
LVEF, %	60 (55–65)	60 (55–63)	60 (56–65)	60 (58–65)	60 (55–65)	35 (30–42)	60 (55–63)	62 (59–65)	64 (58–68)
Computed tomography									
Calcium score, AU^a^	1,620 (876–2,964)	1,916 (1,046–3,366)	1,306 (616–2,320)	878 (415–1,447)	2,941 (1,832–4,291)	1,922 (1,276–3,062)	1,649 (1,034–2,448)	1,066 (650–1,853)	2,434 (1,014–3,842)
Calcium volume, mm^2^	432 (151–915)	557 (236–1,095)	292 (86–644)	148 (50–336)	828 (464–1,437)	467 (242–990)	362 (168–684)	212 (106–478)	599 (329–1,167)
Indexed calcium volume, mm^2^/m^2^	101 (39–199)	120 (52–224)	82 (26–171)	37 (13–75)	194 (115–313)	96 (56–166)	87 (44–150)	57 (28–103)	132 (80–203)
Calcium volume/body surface area, mm^2^/m^2^	236 (86–492)	295 (126–580)	175 (53–376)	82 (30–176)	461 (265–768)	252 (117–484)	193 (92–337)	126 (60–264)	317 (199–482)

Values are %, mean ± SD, or median (Q1-Q3), unless otherwise indicated.

a Calcium score data available for 969 patients.

AU = Agatston Units; LVEF = left ventricular ejection fraction.

**TABLE 2:** Patient Characteristics According to the Primary Endpoint

	All (N = 711)	Primary Endpoint Reached (n = 249)	No Primary Endpoint (n = 462)	*P* Value
Age, y	73 ± 10	74 ± 9	72 ± 11	**0.016**
Male	60	68	56	**0.001**
Body mass index, kg/m^2^	26 ± 5	27 ± 6	26 ± 5	**0.003**
Hypertension	70	74	67	0.088
Hyperlipidemia	40	47	37	**0.007**
Diabetes mellitus	27	30	26	0.160
Current or ex-smoker	32	38	29	**0.020**
Coronary artery disease	20	21	19	0.523
Bicuspid aortic valve	14	12	16	0.178
Peak velocity, m/s	3.3 (2.7–4.0)	3.6 (3.1–4.1)	3.1 (2.6–3.8)	**<0.001**
Mean gradient, mm Hg	24 (16–37)	30 (21–40)	21 (15–33)	**<0.001**
Aortic valve area, cm^2^	1.03 (0.80–1.35)	0.90 (0.74–1.16)	1.14 (0.84–1.43)	**<0.001**
Stroke volume index, mL/m^2^	43 (36–50)	41 (34–48)	44 (37–52)	**<0.001**
LVEF, %	60 (58–65)	60 (57–63)	62 (59–66)	**<0.001**
Discordant aortic stenosis	22	29	18	**<0.001**
Calcium score, AU^a^	1,220 (671–2,126)	1,688 (952–2,825)	1,060 (570–1,761)	**<0.001**
Indexed calcium volume, mm^3^/cm^2^	56 (22–128)	90 (38–174)	41 (15–98)	**<0.001**

Values are mean ± SD, %, or median (Q1-Q3), unless otherwise indicated. **Bold** indicates significant *P* values.

a Calcium score data available in 526 patients.

Abbreviations as in Table 1.

## PERSPECTIVES

### COMPETENCY IN MEDICAL KNOWLEDGE:

Sex-specific indexed aortic valve calcium volume thresholds from CTA provide excellent discrimination for detecting severe aortic stenosis and are independently associated with aortic valve replacement or death.

### TRANSLATIONAL OUTLOOK:

Given the routine use of CTA for both evaluation of coronary artery disease and planning of transcatheter aortic valve implantation, indexed aortic valve calcium volumes could be readily integrated into clinical practice, thereby eliminating the need to assess the calcium score from an additional noncontrast CT scan.

## APPENDIX

For supplemental tables, figures, tables, and references, please see the online version of this paper.

## ABBREVIATIONS AND ACRONYMS

CT: computed tomography
CTA: computed tomography angiography

## References

[1] Nkomo VT, Gardin JM, Skelton TN, Burden of valvular heart diseases: a population-based study. Lancet. 2006;368:1005–1011. 16980116 10.1016/S0140-6736(06)69208-8

[2] Osnabrugge RLJ, Mylotte D, Head SJ, Aortic stenosis in the elderly: disease prevalence and number of candidates for transcatheter aortic valve replacement: a meta-analysis and modeling study. J Am Coll Cardiol. 2013;62:1002–1012. 23727214 10.1016/j.jacc.2013.05.015

[3] Patel A, Kirtane AJ. Aortic valve stenosis. JAMA Cardiol. 2016;1:623. 27434020 10.1001/jamacardio.2016.2060

[4] Braunwald E On the natural history of severe aortic stenosis. J Am Coll Cardiol. 1990;15:1018–1020. 2312955 10.1016/0735-1097(90)90235-h

[5] Clavel MA, Messika-Zeitoun D, Pibarot P, The complex nature of discordant severe calcified aortic valve disease grading: new insights from combined Doppler echocardiographic and computed tomographic study. J Am Coll Cardiol. 2013;62:2329–2338. 24076528 10.1016/j.jacc.2013.08.1621

[6] Clavel MA, Pibarot P, Messika-Zeitoun D, Impact of aortic valve calcification, as measured by MDCT, on survival in patients with aortic stenosis: results of an international registry study. J Am Coll Cardiol. 2014;64:1202–1213. 25236511 10.1016/j.jacc.2014.05.066 PMC4391203

[7] Pawade T, Clavel MA, Tribouilloy C, Computed tomography aortic valve calcium scoring in patients with aortic stenosis. Circ Cardiovasc Imaging. 2018;11(3):e007146. 29555836 10.1161/CIRCIMAGING.117.007146

[8] Francone M, Budde RPJ, Bremerich J, CT and MR imaging prior to transcatheter aortic valve implantation: standardisation of scanning protocols, measurements and reporting—a consensus document by the European Society of Cardiovascular Radiology (ESCR). Eur Radiol. 2020;30:2627–2650. 31489471 10.1007/s00330-019-06357-8 PMC7160220

[9] Narula J, Chandrashekhar Y, Ahmadi A, SCCT 2021 Expert Consensus Document on Coronary Computed Tomographic Angiography: A Report of the Society of Cardiovascular Computed Tomography. J Cardiovasc Comput Tomogr. 2021;15:192–217. 33303384 10.1016/j.jcct.2020.11.001 PMC8713482

[10] Writing Committee Members, Otto CM, Nishimura RA, 2020 ACC/AHA Guideline for the Management of Patients With Valvular Heart Disease: A Report of the American College of Cardiology/American Heart Association Joint Committee on Clinical Practice Guidelines. J Am Coll Cardiol. 2021;77:e25–e197. 33342586 10.1016/j.jacc.2020.11.018

[11] Baumgartner H, Hung J, Bermejo J, Recommendations on the echocardiographic assessment of aortic valve stenosis: a focused update from the European Association of Cardiovascular Imaging and the American Society of Echocardiography. J Am Soc Echocardiogr. 2017;30:372–392. 28385280 10.1016/j.echo.2017.02.009

[12] Baumgartner H, Hung J, Bermejo J, Echocardiographic assessment of valve stenosis: EAE/ASE recommendations for clinical practice. J Am Soc Echocardiogr. 2009;22:1–23. 19130998 10.1016/j.echo.2008.11.029

[13] Dweck MR, Loganath K, Bing R, Multi-modality imaging in aortic stenosis: an EACVI clinical consensus document. Eur Heart J Cardiovasc Imaging. 2023;24:1430–1443. 37395329 10.1093/ehjci/jead153

[14] Vahanian A, Beyersdorf F, Praz F, 2021 ESC/EACTS guidelines for the management of valvular heart disease. Eur Heart J. 2022;43:561–632. 34453165 10.1093/eurheartj/ehab395

[15] Pawade T, Sheth T, Guzzetti E, Why and how to measure aortic valve calcification in patients with aortic stenosis. JACC Cardiovasc Imaging. 2019;12:1835–1848. 31488252 10.1016/j.jcmg.2019.01.045

[16] Oliveira DAB, Feitosa RQ, Correia MM. Segmentation of liver, its vessels and lesions from CT images for surgical planning. Biomed Eng Online. 2011;10:30. 21507229 10.1186/1475-925X-10-30 PMC3094217

[17] Dey D, Cheng VY, Slomka PJ, Automated 3-dimensional quantification of non-calcified and calcified coronary plaque from coronary CT angiography. J Cardiovasc Comput Tomogr. 2009;3:372–382. 10.1016/j.jcct.2009.09.004 20083056

[18] Patel KP, Lin A, Kumar N, Influence of cusp morphology and sex on quantitative valve composition in severe aortic stenosis. Eur Hear J Cardiovasc Imaging. 2023;24:1653–1660. 10.1093/ehjci/jead142 37339331

[19] Blanke P, Weir-McCall JR, Achenbach S, Computed tomography imaging in the context of transcatheter aortic valve implantation (TAVI)/transcatheter aortic valve replacement (TAVR): an expert consensus document of the Society of Cardiovascular Computed Tomography. JACC Cardiovasc Imaging. 2019;12:1–24. 30621986 10.1016/j.jcmg.2018.12.003

[20] Minners J, Allgeier M, Gohlke-Baerwolf C, Inconsistent grading of aortic valve stenosis by current guidelines: haemodynamic studies in patients with apparently normal left ventricular function. Heart. 2010;96:1463–1468. 20813727 10.1136/hrt.2009.181982

[21] Messika-Zeitoun D, Aubry MC, Detaint D, Evaluation and clinical implications of aortic valve calcification measured by electron-beam computed tomography. Circulation. 2004;110:356–362. 15249504 10.1161/01.CIR.0000135469.82545.D0

[22] Simard L, Côté N, Dagenais F, Sex-related discordance between aortic valve calcification and hemodynamic severity of aortic stenosis. Circ Res. 2017;120:681–691. 27879282 10.1161/CIRCRESAHA.116.309306

[23] Cartlidge TRG, Bing R, Kwiecinski J, Contrast-enhanced computed tomography assessment of aortic stenosis. Heart. 2021;107:1905–1911. 33514522 10.1136/heartjnl-2020-318556 PMC8600609

