# Supplementary material for: Indexed Aortic Valve Calcium Volume by Computed Tomography Angiography in Patients With Aortic Stenosis: Results of an International Multicenter Cohort Study
Source: JACC Cardiovasc Imaging. Author manuscript; Available in PMC 2026 Apr 30. (PMC13132100; doi:10.1016/j.jcmg.2025.09.013)
Supplement: supplement [file NIHMS2136147-supplement-supplement.docx]

**
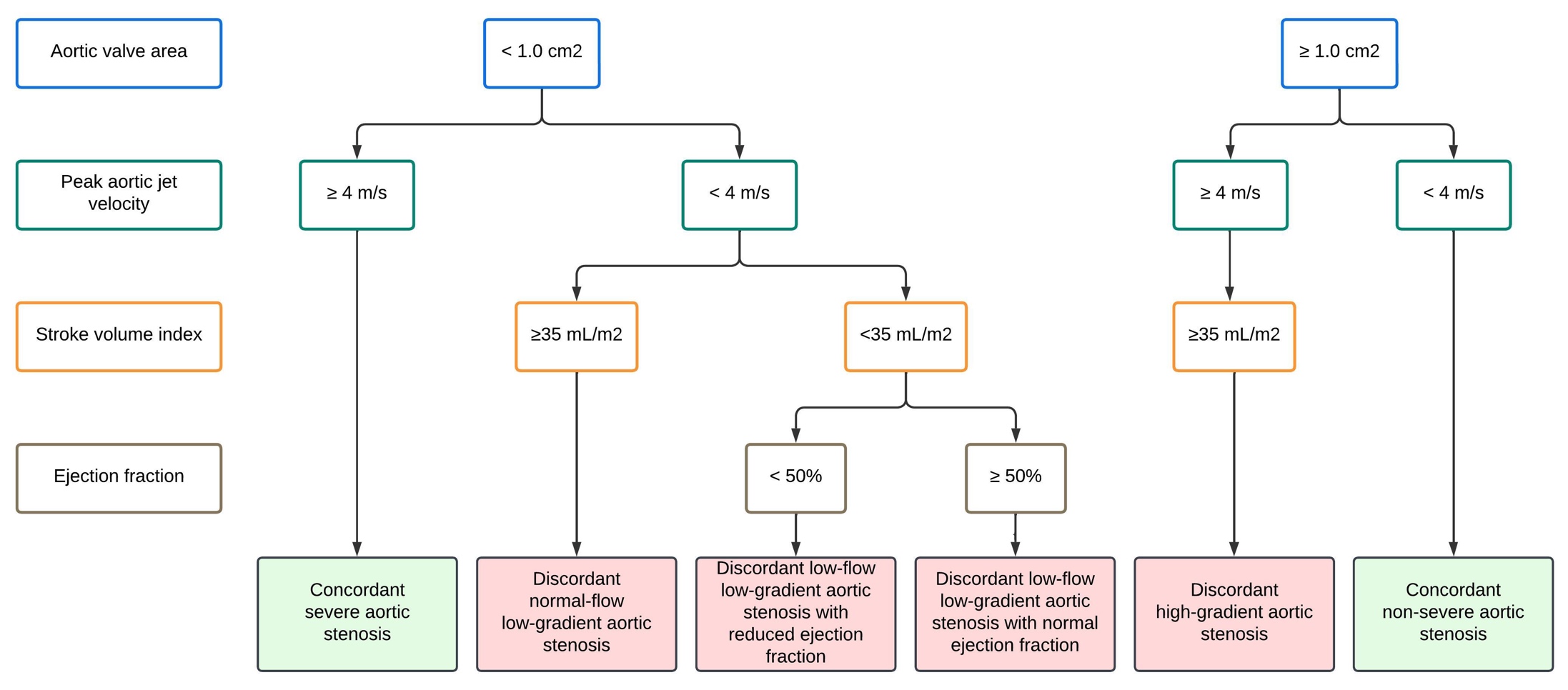
**

**Supplemental Figure 1**

Overview of the echocardiographic classification of aortic stenosis severity


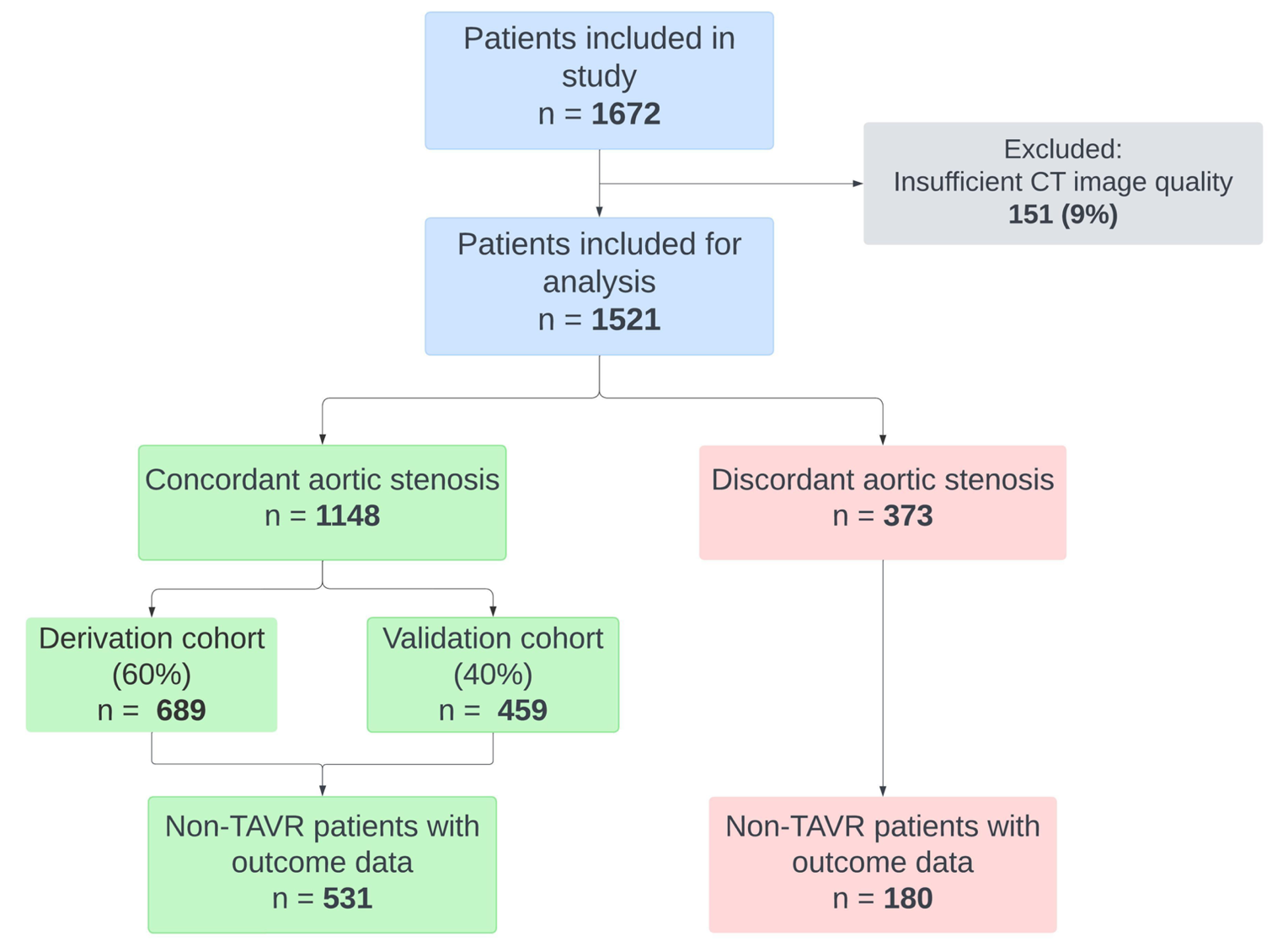


**Supplemental Figure 2**

Flowchart of the study population

CT = computed tomography, TAVR = transcatheter aortic valve replacement.


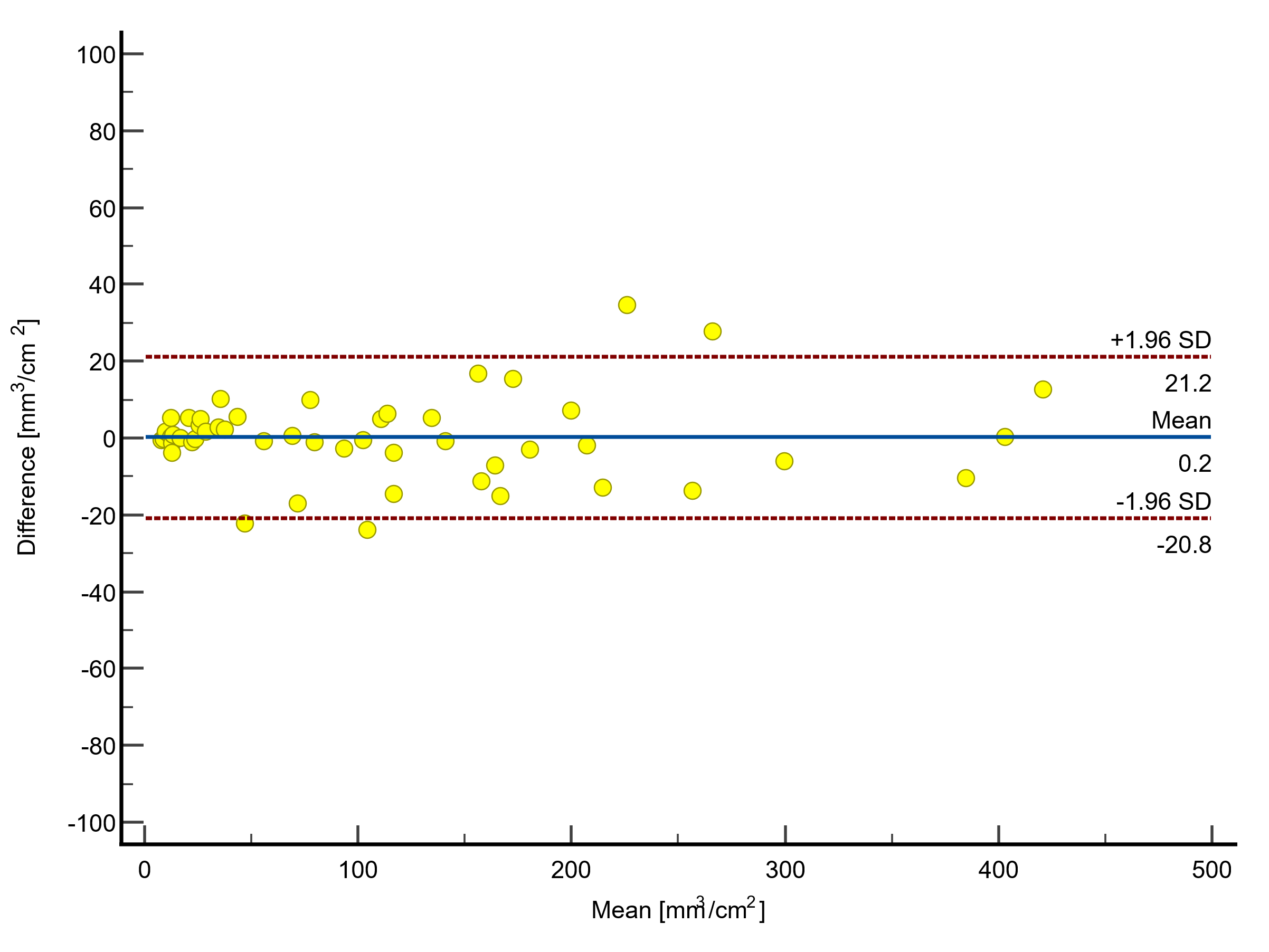

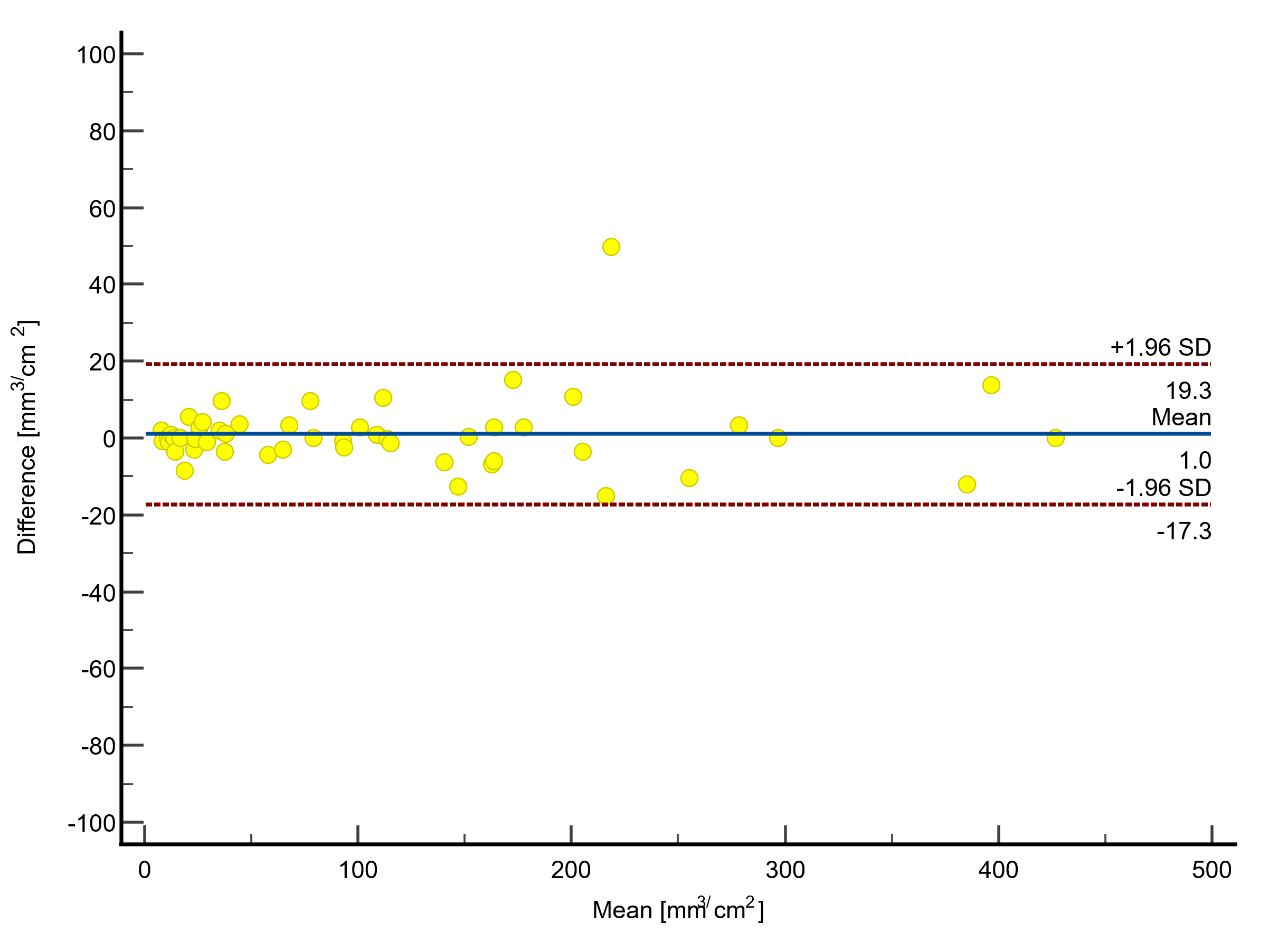

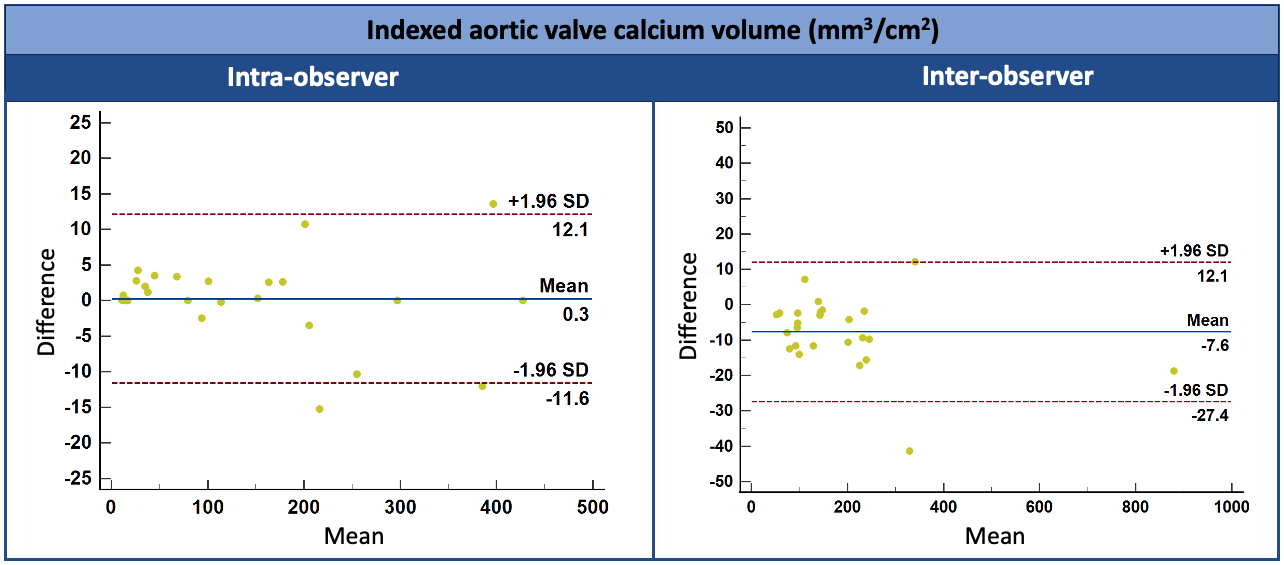


Difference

Mean

Mean

Difference

**Supplemental Figure 3**

Bland Altman plots for intra-observer and inter-observer reproducibility for calcific volume and calcific volume indexed to annulus area

The dotted red lines represent 95% confidence interval limits of agreement and the solid blue lines represent mean difference.

**
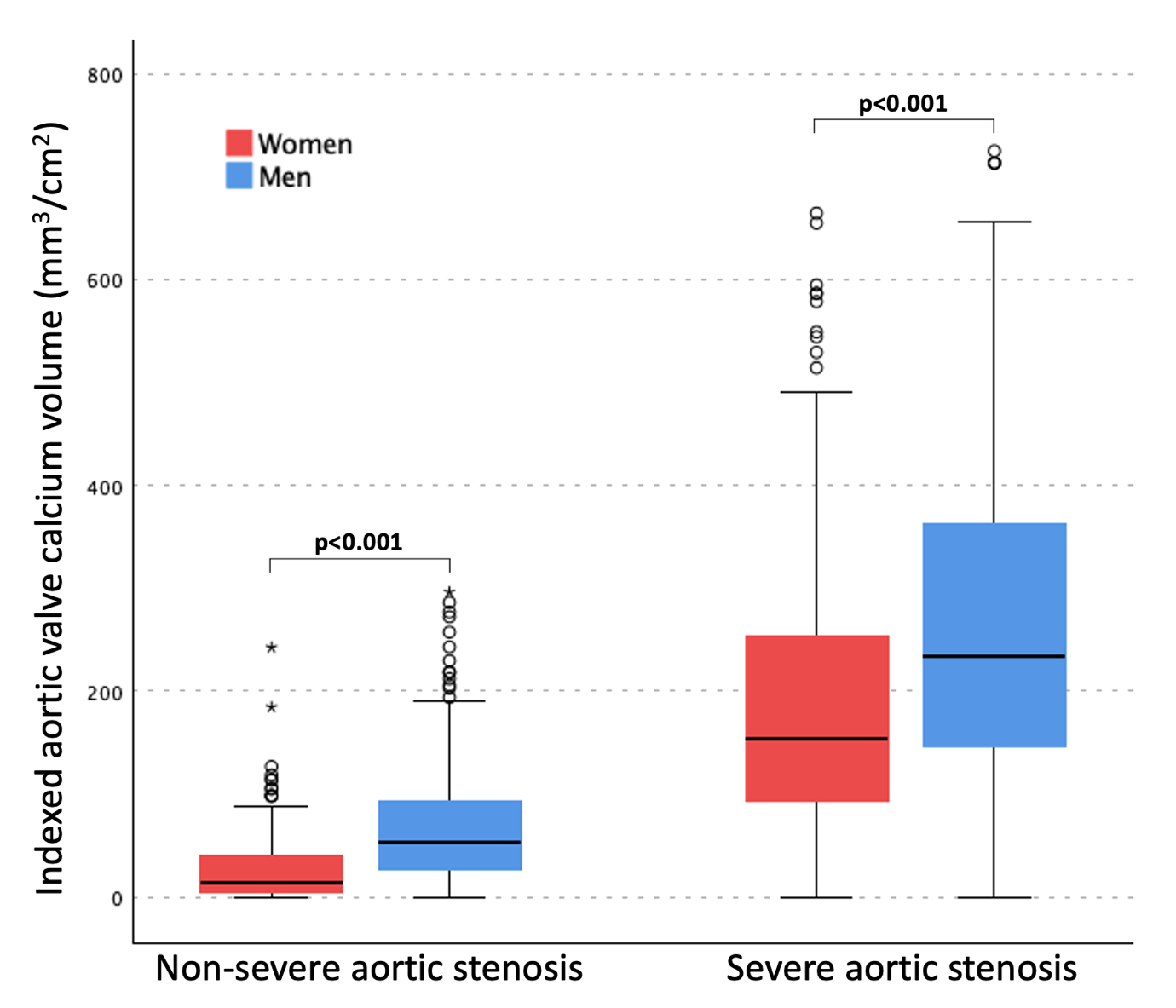
**

**Supplemental Figure 4**

Indexed aortic valve calcium volume in patients with concordant echocardiographic disease according to sex and aortic stenosis severity

**
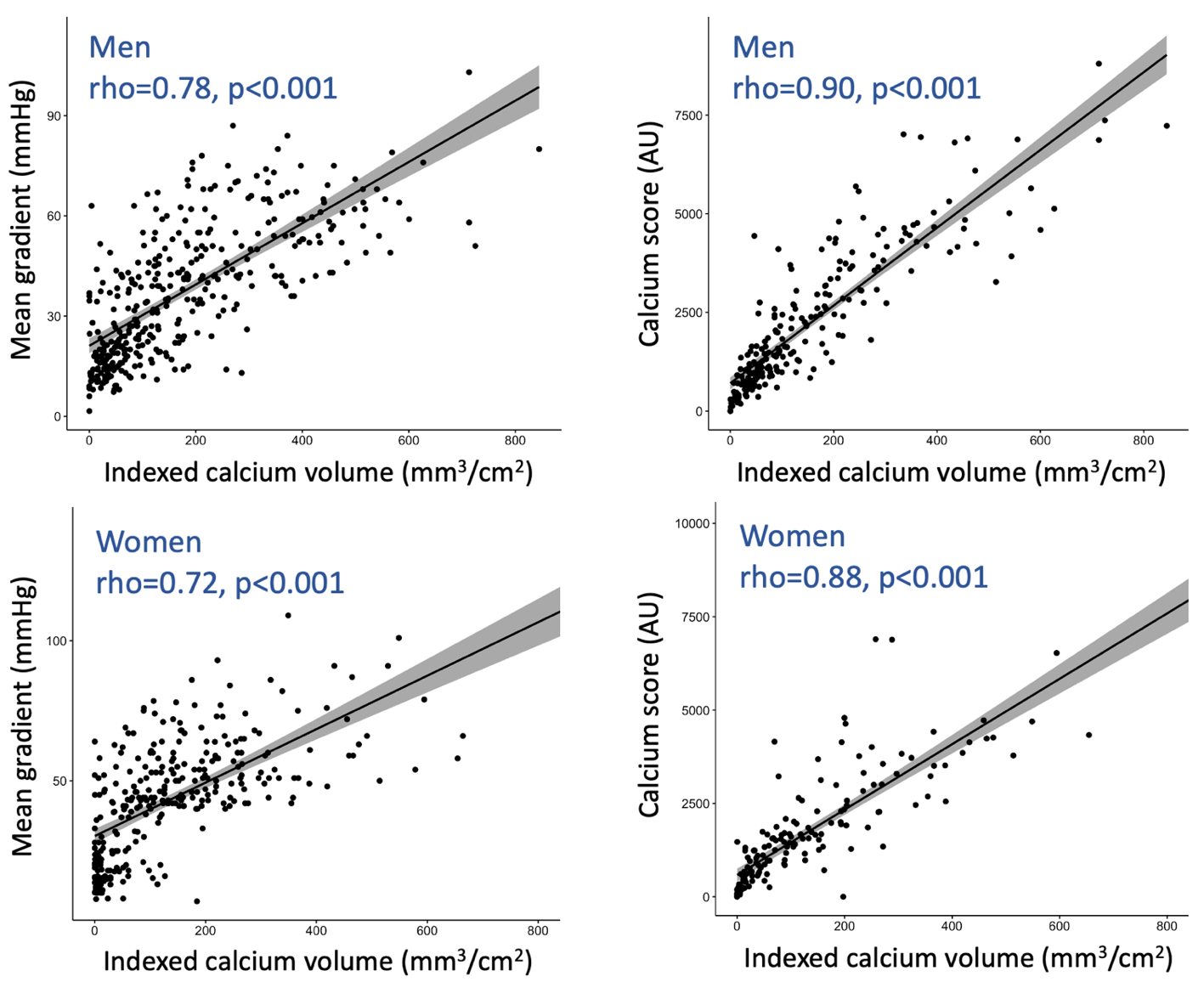
**

**Supplemental Figure 5**

Correlation between aortic valve calcific volume and aortic stenosis disease severity as assessed by echocardiography and non-contrast computed tomography (CT) calcium score

**
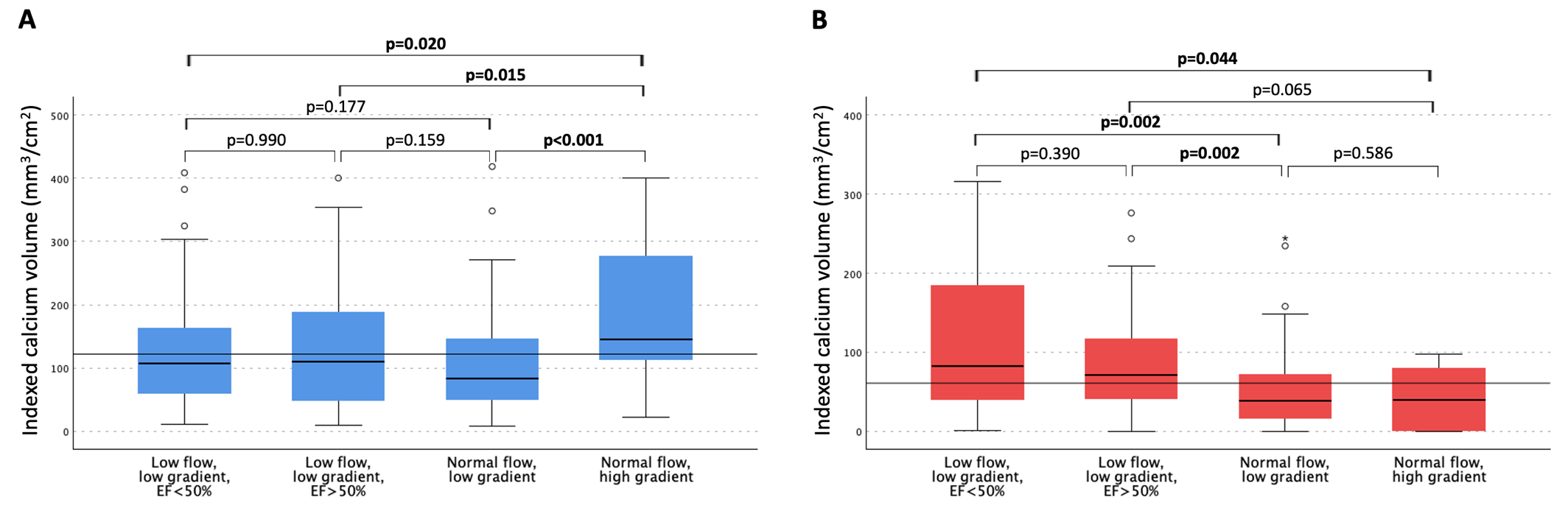
**

**Supplemental Figure 6**

Distribution of indexed aortic valve calcium volume in male (**A**) and female (**B**) patients with discordant grading on echocardiography

A score above the black line represents indexed calcium volume above the sex-specific threshold and therefore severe aortic valve calcification; a score below the black line and sex-specific threshold represents non-severe calcification. Important heterogeneity in aortic valve calcification was observed in both male and female patients with discordant grading on echocardiography. Box and whiskers plot error bars are from the 10^th^ and 90^th^ centile and the horizontal line represents the median value. EF = ejection fraction.

**
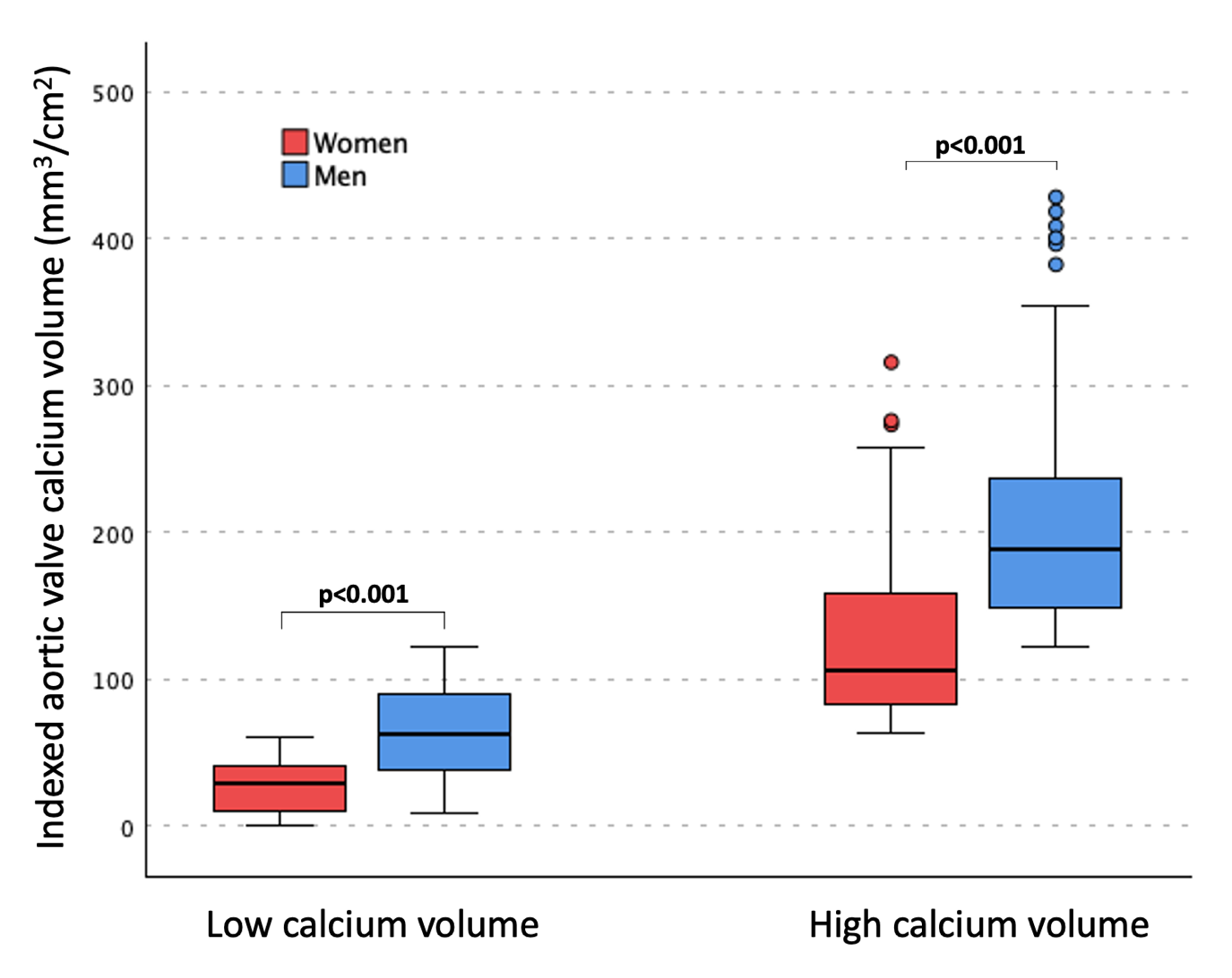
**

**Supplemental Figure 7**

Indexed aortic valve calcium volume in patients with discordant echocardiographic disease according to sex and aortic stenosis severity defined by the sex-specific indexed aortic valve calcium volume threshold (122 mm^3^/cm^2^ in men and 61 mm^3^/cm^2^ in women)

**Supplemental Table 1**

Overview and specifications of participating centers

| CENTRE | COHORT | CT SCANNER | NUMBER OF SCANS |
| --- | --- | --- | --- |
| British Heart Foundation Centre of Research Excellence, University of Edinburgh, United Kingdom | Research: SALTIRE2, SALTIRE3 | Biograph mCT, Siemens Medical Systems | 205 |
| Guy’s and St Thomas’ NHS Foundation Trust, London, United Kingdom | Research | Brilliance iCT, Philips Healthcare  SOMATOM Force, Siemens Medical Systems | 67 |
| Barts Health NHS Trust, London, United Kingdom | Research | SOMATOM Force, Siemens Medical Systems | 31 |
| Hospital Vall d’Hebron, Barcelona, Spain | Research | GE Discovery CT750 HD, GE Healthcare  Brilliance iCT 256, Philips Healthcare | 52 |
| Odense University Hospital, Odense, Denmark | Research: DALLAS | GE Revolution, GE Healthcare | 47 |
| Medical University of Gdansk, Gdansk, Poland | Clinical | SOMATOM Definition Flash, Siemens Medical systems  GE Revolution, GE Healthcare | 27 |
| POL-TAVI registry including:  Medical University of Gdansk, Gdansk; Medical University of Lodz, Lodz;  Medical University of Poznan, Poznan; Medical University of Silesia, Katowice; Medical University of Warsaw, Warsaw; Wroclaw Medical University, Wroclaw | Clinical | SOMATOM Definition Flash, Siemens Medical systems  GE Revolution, GE Healthcare  Aquilion ONE, Toshiba Medical Systems  GE Discovery CT750 HD, GE Healthcare | 415 |
| Seoul National University Hospital, Seoul, South Korea | Clinical | SOMATOM Force & SOMATOM Definition Flash & Sensation 16, Siemens Medical Systems  GE Revolution & GE Discovery CT750 HD, GE Healthcare  Aquilion ONE, Toshiba Medical Systems  Brilliance iCT 256, Philips Healthcare | 517 |
| Asan Medical Center Heart Institute, Seoul, South Korea | Clinical | SOMATOM Definition Flash & SOMATOM Definition AS+ & SOMATOM Force, Siemens Medical Systems  GE Lightspeed VCT & GE Discovery CT750 HD, GE Healthcare | 240 |
| Cedars-Sinai Smidt Heart Institute, Los Angeles, California, USA | Clinical | Siemens Definition, Definition Flash | 71 |

**Supplemental Table 2** STROBE Statement – checklist of items that should be included in reports of observational studies

|  | | Item No. | Recommendation | Page  No. | | Relevant text from manuscript |
| --- | --- | --- | --- | --- | --- | --- |
| **Title and abstract** | | 1 | (*a*) Indicate the study’s design with a commonly used term in the title or the abstract | 1,4 | |  |
|  |  |  | (*b*) Provide in the abstract an informative and balanced summary of what was done and what was found | 4,5 | |  |
| Introduction | | | | | |  |
| Background/rationale | | 2 | Explain the scientific background and rationale for the investigation being reported | 6,7 | |  |
| Objectives | | 3 | State specific objectives, including any prespecified hypotheses | 7 | |  |
| Methods | | | | | |  |
| Study design | | 4 | Present key elements of study design early in the paper | 8 | |  |
| Setting | | 5 | Describe the setting, locations, and relevant dates, including periods of recruitment, exposure, follow-up, and data collection | 8-11 | |  |
| Participants | | 6 | (*a*) *Cohort study*—Give the eligibility criteria, and the sources and methods of selection of participants. Describe methods of follow-up  *Case-control study*—Give the eligibility criteria, and the sources and methods of case ascertainment and control selection. Give the rationale for the choice of cases and controls  *Cross-sectional study*—Give the eligibility criteria, and the sources and methods of selection of participants | 8,11 | |  |
|  |  |  | (*b*) *Cohort study*—For matched studies, give matching criteria and number of exposed and unexposed  *Case-control study*—For matched studies, give matching criteria and the number of controls per case |  | |  |
| Variables | | 7 | Clearly define all outcomes, exposures, predictors, potential confounders, and effect modifiers. Give diagnostic criteria, if applicable | 11,12 | |  |
| Data sources/ measurement | | 8 | For each variable of interest, give sources of data and details of methods of assessment (measurement). Describe comparability of assessment methods if there is more than one group | *8-12* | |  |
| Bias | | 9 | Describe any efforts to address potential sources of bias | 10-12 | |  |
| Study size | | 10 | Explain how the study size was arrived at | 13 | |  |
| Quantitative variables | | 11 | Explain how quantitative variables were handled in the analyses. If applicable, describe which groupings were chosen and why | 11,12 | |  |
| Statistical methods | | 12 | (a) Describe all statistical methods, including those used to control for confounding | 11,12 | |  |
|  | |  | (b) Describe any methods used to examine subgroups and interactions | 11,12 | |  |
|  | |  | (c) Explain how missing data were addressed |  | |  |
|  | |  | (d) Cohort study—If applicable, explain how loss to follow-up was addressed  Case-control study—If applicable, explain how matching of cases and controls was addressed  Cross-sectional study—If applicable, describe analytical methods taking account of sampling strategy | 11,15,16 | |  |
|  | |  | (e) Describe any sensitivity analyses |  | |  |
| Results | | | | | | |
| Participants | | 13 | (a) Report numbers of individuals at each stage of study—eg numbers potentially eligible, examined for eligibility, confirmed eligible, included in the study, completing follow-up, and analysed | 13-16 |  | |
|  |  |  | (b) Give reasons for non-participation at each stage | 13-16 |  | |
|  |  |  | (c) Consider use of a flow diagram | Supplementary Figure 2 |  | |
| Descriptive data | | 14 | (a) Give characteristics of study participants (eg demographic, clinical, social) and information on exposures and potential confounders | 13, Table 1 |  | |
|  |  |  | (b) Indicate number of participants with missing data for each variable of interest |  |  | |
|  |  |  | (c) *Cohort study*—Summarise follow-up time (eg, average and total amount) | 15,16 |  | |
| Outcome data | | 15 | *Cohort study*—Report numbers of outcome events or summary measures over time | 15,16 |  | |
|  |  |  | *Case-control study—*Report numbers in each exposure category, or summary measures of exposure |  |  | |
|  |  |  | *Cross-sectional study—*Report numbers of outcome events or summary measures |  |  | |
| Main results | | 16  17 | (*a*) Give unadjusted estimates and, if applicable, confounder-adjusted estimates and their precision (eg, 95% confidence interval). Make clear which confounders were adjusted for and why they were included | 15,16, Supplementary Table 9 & 10 |  | |
|  |  |  | (*b*) Report category boundaries when continuous variables were categorized | 9,14 |  | |
|  |  |  | (*c*) If relevant, consider translating estimates of relative risk into absolute risk for a meaningful time period |  |  | |
|  |  |  | Report other analyses done—eg analyses of subgroups and interactions, and sensitivity analyses | 13-16 |  | |
| Discussion | | | | | | |
| Key results |  | 18 | Summarise key results with reference to study objectives | 17 | | |
| Limitations |  | 19 | Discuss limitations of the study, taking into account sources of potential bias or imprecision. Discuss both direction and magnitude of any potential bias | 19,20 | | |
| Interpretation |  | 20 | Give a cautious overall interpretation of results considering objectives, limitations, multiplicity of analyses, results from similar studies, and other relevant evidence | 17-20 | | |
| Generalisability |  | 21 | Discuss the generalisability (external validity) of the study results | 17-20 | | |
| Other information | |  | | | | |
| Funding |  | 22 | Give the source of funding and the role of the funders for the present study and, if applicable, for the original study on which the present article is based | 21 | | |

**Supplemental Table 3**

Uni- and multivariable associations of clinical variables with indexed aortic valve calcium volume

|  | Univariable | | | Multivariable | | |
| --- | --- | --- | --- | --- | --- | --- |
|  | B coefficient | 95% Confidence Interval | P-value | B coefficient | 95% Confidence Interval | P-value |
| Age | 2.8 | 2.2 – 3.4 | <0.001 | 3.0 | 2.3 – 3.8 | <0.001 |
| Male | 39.6 | 25.9 – 53.7 | <0.001 | 51.2 | 36.1 – 66.3 | <0.001 |
| Body-mass index | 0.7 | -8 – 2.3 | 0.347 | - | - | - |
| Hypertension | 25.0 | 9.6 – 40.5 | 0.002 | - | - | - |
| Hyperlipidemia | 34.9 | 20.9 – 48.9 | <0.001 | - | - | - |
| Diabetes Mellitus | 9.8 | -5.7 – 25.4 | 0.215 | - | - | - |
| Coronary artery disease | 50.6 | 33.9 – 67.3 | <0.001 | 40.2 | 22.6 – 57.8 | <0.001 |
| Bicuspid aortic valve | 32.8 | 13.8 – 51.8 | <0.001 | 96.4 | 75.5 – 117.3 | <0.001 |
| Left ventricular ejection fraction | -1.5 | -2.2 - -0.8 | <0.001 | -1.3 | -2.1 - -0.5 | 0.003 |

**Supplemental Table 4**

Patient characteristics of White and Asian populations

|  | | Concordant  non-severe aortic stenosis | | | Concordant severe aortic stenosis | | |
| --- | --- | --- | --- | --- | --- | --- | --- |
|  |  | White | Asian | p-value | White | Asian | p-value |
| Clinical | | 206 | 281 |  | 400 | 261 |  |
| Age | y | 71±12 | 71±11 | 0.965 | 79±8 | 71±11 | **<0.001** |
| Male | % | 71 | 57 | **0.001** | 48 | 55 | 0.080 |
| Body-mass index | kg/m^2^ | 29±5 | 24±4 | **<0.001** | 27±4 | 24±4 | **<0.001** |
| Hypertension | % | 75 | 66 | 0.067 | 79 | 56 | **<0.001** |
| Hyperlipidemia | % | 62 | 33 | **<0.001** | 82 | 18 | **<0.001** |
| Diabetes Mellitus | % | 22 | 30 | 0.068 | 35 | 23 | **0.001** |
| Current or ex-smoker | % | 52 | 20 | **<0.001** | 36 | 23 | 0.055 |
| Coronary artery disease | % | 26 | 13 | **0.001** | 72 | 6 | **<0.001** |
| Bicuspid aortic valve | % | 15 | 10 | 0.110 | 12 | 38 | **<0.001** |
| Echocardiography | | | | | | | |
| Peak velocity | m/s | 3.0 (2.5 – 3.3) | 3.1 (2.8 – 3.6) | 0.967 | 4.4 (4.2 – 4.9) | 4.6 (4.3 – 4.6) | **0.002** |
| Mean gradient | mmHg | 18 (13 – 23) | 22 (17 – 28) | 0.773 | 48 (42 – 57) | 52 (43 – 66) | **<0.001** |
| Aortic valve area | cm^2^ | 1.26 (1.13 – 1.49) | 1.20 (1.07 – 1.37) | 0.212 | 0.67 (0.50 – 0.80) | 0.65 (0.56 – 0.77) | 0.199 |
| Stroke volume | mL/m^2^ | 42 (37 – 51) | 46 (40 – 55) | **<0.001** | 41 (36 – 51) | 45 (39 – 53) | **0.007** |
| Ejection fraction | % | 60 (58 – 60) | 63 (59 – 67) | **<0.001** | 58 (54 – 60) | 63 (59 – 66) | **<0.001** |
| Computed Tomography | | | | | | | |
| Calcium score* | AU | 817 (428 – 1432) | 969 (398 – 1489) | 0.483 | 3085 (1999 – 4242) | 2466 (1514 – 4375) | 0.067 |
| Indexed calcium volume | mm^3^/cm^2^ | 40 (15 – 87) | 50 (19 – 89) | **0.029** | 217 (131 – 329) | 155 (91 – 263) | **<0.001** |

Significant p-values are highlighted in bold. * Calcium scores available in 314 patients with non-severe and 344 patients with severe aortic stenosis

**Supplemental Table 5**

Multivariable associations of clinical variables and ethnicity with indexed aortic valve calcium volume

|  | Concordant non-severe aortic stenosis | | | Concordant severe aortic stenosis | | |
| --- | --- | --- | --- | --- | --- | --- |
|  | B coefficient | 95% Confidence Interval | P-value | B coefficient | 95% Confidence Interval | P-value |
| Asian | - | - | - | - | - | - |
| Age | 0.8 | 0.3 – 1.4 | <0.001 | 3.4 | 1.9 – 4.8 | <0.001 |
| Male | 38.4 | 28.0 – 48.8 | <0.001 | 92.9 | 65.2 – 120.6 | <0.001 |
| Body-mass index | - | - | - | - | - | - |
| Hypertension | - | - | - | - | - | - |
| Hyperlipidemia | - | - | - | - | - | - |
| Diabetes Mellitus | - | - | - | -32.5 | -63.2 - -1.8 | 0.038 |
| Coronary artery disease | - | - | - | 53.8 | 22.1 – 85.4 | 0.001 |
| Bicuspid aortic valve | 25.7 | 8.2 – 43.1 | 0.004 | 64.8 | 30.5 – 99.0 | <0.001 |

**Supplemental Table 6**

Intra- and inter-observer repeatability measurements

|  | **Mean difference** | **Upper limit of agreement** | **Lower limit of agreement** | **Mean absolute difference** | **Standard error of the mean** | **Correlation coefficient** |
| --- | --- | --- | --- | --- | --- | --- |
|  | **Intra-observer repeatability** | | | | | |
| Indexed aortic valve calcium volume (mm^3^/cm^2^) | 1.0 | 19.3 | -17.3 | 5.27 | 1.31 | 0.998 |
|  | **Inter-observer repeatability** | | | | | |
| Indexed aortic valve calcium volume (mm^3^/cm^2^) | 0.2 | 21.2 | -20.8 | 16.59 | 7.96 | 0.996 |

**Supplemental Table 7**

Correlation between aortic valve calcium and aortic stenosis disease severity on echocardiography

|  | | Aortic Valve Mean Gradient (mmHg) | | | Aortic Valve Peak Velocity (m/s) | | | Calcium Score (Agatston Units) | | |
| --- | --- | --- | --- | --- | --- | --- | --- | --- | --- | --- |
|  | | Overall | Men | Women | Overall | Men | Women | Overall | Men | Women |
| Calcium score  (Agatston Units) | Correlation | 0.714 | 0.771 | 0.731 | 0.711 | 0.755 | 0.728 |  |  |  |
|  | P value | **<0.001** | **<0.001** | **<0.001** | **<0.001** | **<0.001** | **<0.001** |  |  |  |
| Indexed calcium volume  (mm^3^/cm^2^) | Correlation | 0.722 | 0.775 | 0.724 | 0.723 | 0.755 | 0.715 | 0.896 | 0.904 | 0.881 |
|  | P value | **<0.001** | **<0.001** | **<0.001** | **<0.001** | **<0.001** | **<0.001** | **<0.001** | **<0.001** | **<0.001** |

**Supplemental Table 8**

Patient characteristics of the derivation cohort

|  | | All | Men | Women | Non-severe | Severe |
| --- | --- | --- | --- | --- | --- | --- |
| Clinical | | 689 | 384 | 305 | 274 | 415 |
| Age | y | 74±11 | 72±11 | 75±10 | 71±11 | 75±10 |
| Male | % | 56 | 100 | 0 | 66 | 49 |
| Body-mass index | kg/m^2^ | 26±5 | 26±5 | 26±5 | 26±5 | 26±5 |
| Hypertension | % | 70 | 70 | 71 | 72 | 69 |
| Hyperlipidemia | % | 49 | 47 | 52 | 41 | 54 |
| Diabetes Mellitus | % | 28 | 30 | 25 | 26 | 29 |
| Current or ex-smoker | % | 28 | 41 | 9 | 33 | 23 |
| Coronary artery disease | % | 26 | 29 | 21 | 20 | 32 |
| Bicuspid aortic valve | % | 19 | 19 | 20 | 14 | 23 |
| Echocardiography | | | | | | |
| Peak velocity | m/s | 4.2 [3.1-4.8] | 4.0 [3.0-4.7] | 4.3 [3.4-4.9] | 2.9 [2.5-3.3] | 4.6 [4.2-5.1] |
| Mean gradient | mmHg | 41 [21-54] | 36 [19-52] | 44 [25-58] | 18 [14-24] | 51 [43-63] |
| Aortic valve area | cm^2^ | 0.80 [0.64-1.20] | 0.94 [0.70-1.29] | 0.75 [0.60-1.07] | 1.29 [1.13-1.50] | 0.68 [0.55-0.78] |
| Ejection fraction | % | 60 [56-65] | 60 [57-64] | 60 [56-66] | 60 [59-65] | 60 [55-65] |
| Computed Tomography | | | | | | |
| Calcium score* | AU | 1489 [765-3048] | 1604 [877-3503] | 1370 [606-2552] | 878 [428-1454] | 2737 [1660-4241] |
| Indexed calcium volume | mm^3^/cm^2^ | 122 [46-234] | 128 [53-258] | 108 [32-217] | 40 [13-86] | 199 [121-317] |

* Calcium scores available in 387 patients.

**Supplemental Table 9**

Performance and optimal thresholds of calcific volume and calcific volume indexed to annulus area to identify severe aortic stenosis in the different cohorts of patients with concordant aortic stenosis

|  |  | **C-statistic** | **Threshold** | **Sensitivity (%)** | **Specificity (%)** |
| --- | --- | --- | --- | --- | --- |
| Indexed calcium volume (mm^3^/cm^2^) | | | | | |
| **Derivation cohort** All patients (n=689) | Men | 0.900 | 122 | 85 | 82 |
|  | Women | 0.926 | 61 | 86 | 87 |
| **Derivation cohort**  Subgroup with calcium score (n=387) | Men | 0.887 | 122 | 81 | 82 |
|  | Women | 0.934 | 63 | 89 | 85 |
| **Validation cohort**  (n=459) | Men | 0.933 | 122 | 83 | 87 |
|  | Women | 0.944 | 67 | 86 | 92 |
|  |  |  | 61* | 87 | 91 |

* Performance of optimal threshold as determined in derivation cohort.

**Supplemental Table 10**

Univariable and multivariable cox regression analysis for the prediction of aortic valve replacement and all-cause mortality in the total study cohort

|  | **Univariable** | | **Multivariable Model 1** | | **Multivariable Model 2** | | **Multivariable Model 3** | |
| --- | --- | --- | --- | --- | --- | --- | --- | --- |
|  | **HR (95% CI)** | **p-value** | **HR (95% CI)** | **p-value** | **HR (95% CI)** | **P Value** | **HR (95% CI)** | **P Value** |
| Age | 1.04  (1.03 to 1.06) | **<0.0001** | 1.02  (0.99 to 1.04) | 0.13 | 1.03  (1.00 to 1.06) | 0.04 | 1.02  (0.99 to 1.04) | 0.08 |
| Sex | 1.35  (1.04 to 1.77) | **0.03** | 1.04  (0.73 to 1.51) | 0.82 | 1.70  (1.10 to 2.65) | 0.02 | 1.08  (0.76 to 1.56) | 0.67 |
| Body-mass index | 1.04 (1.00 to 1.07) | **0.04** | 1.00  (0.96 to 1.04) | 0.95 | 1.00  (0.96 to 1.04) | 0.92 | 1.00  (0.96 to 1.04) | 0.97 |
| Hypertension | 1.43  (1.08 to 1.92) | **0.01** | 1.15  (0.78 to 1.70) | 0.49 | 1.13  (0.72 to 1.82) | 0.60 | 1.15  (0.78 to 1.71) | 0.48 |
| Smoking history | 1.50  (1.12 to 1.98) | **<0.01** | 1.34  (0.89 to 2.00) | 0.15 | 1.33  (0.83 to 2.18) | 0.23 | 1.34  (0.89 to 2.00) | 0.16 |
| Hyperlipidaemia | 1.61  (1.25 to 2.08) | **<0.001** | 1.31  (0.92 to 1.85) | 0.14 | 1.30  (0.85 to 1.97) | 0.21 | 1.33  (0.93 to 1.89) | 0.12 |
| Diabetes | 1.40  (1.06 to 1.83) | **0.01** | 1.41  (0.99 to 1.99) | 0.05 | 0.99  (0.64 to 1.49) | 0.96 | 1.37  (0.96 to 1.93) | 0.08 |
| Chronic Kidney Disease | 2.40  (1.62 to 3.44) | **<0.0001** | 2.24  (1.31 to 3.65) | **<0.01** | 1.48  (0.76 to 2.66) | 0.22 | 2.02  (1.19 to 3.28) | **<0.01** |
| Coronary Artery Disease | 1.42  (1.03 to 1.91) | **0.03** | 1.00  (0.64 to 1.53) | 0.99 | 1.02  (0.59 to 1.69) | 0.94 | 0.99  (0.63 to 1.51) | 0.96 |
| Bicuspid Aortic Valve Disease | 0.64  (0.43 to 0.92) | **0.02** | 0.69  (0.41 to 1.12) | 0.15 | 0.60  (0.29 to 1.17) | 0.15 | 0.73  (0.43 to 1.18) | 0.21 |
| Left Ventricular Ejection Fraction | 0.26  (0.78 to 0.38) | **<0.0001** | 0.28  (0.17 to 0.48) | <0.0001 | 0.21  (0.12 to 0.42) | **<0.0001** | 0.27  (0.16 to 0.47) | **<0.0001** |
| Peak aortic jet-velocity^a^ | 4.02  (2.85 to 5.68) | **<0.0001** | 2.20  (1.23 to 3.88) | <0.01 | 1.50  (1.16 to 1.92) | **<0.01** | 2.96  (1.68 to 5.28) | **<0.001** |

|  | **Univariable** | | **Multivariable Model 1** | | **Multivariable Model 2** | | **Multivariable Model 3** | |
| --- | --- | --- | --- | --- | --- | --- | --- | --- |
|  | **HR (95% CI)** | **p-value** | **HR (95% CI)** | **p-value** | **HR (95% CI)** | **P Value** | **HR (95% CI)** | **P Value** |
| High indexed calcium volume | 2.64  (2.05 to 3.40) | **<0.0001** | 2.01  (1.30 to 3.10) | **<0.01** |  |  |  |  |
| High calcium score | 2.42  (1.83 to 3.21) | **<0.0001** |  |  | 1.38  (0.86 to 2.19) | 0.18 |  |  |
| High non-indexed calcium volume | 2.36  (1.82 to 3.04) | **<0.0001** |  |  |  |  | 1.37  (0.86 to 2.15) | 0.18 |

^a^ log_2_ transformation was applied to achieve normal distribution. HR = hazard ratio.

**Supplemental Table 11**

Univariable and multivariable cox regression analysis for the prediction of aortic valve replacement and all-cause mortality in patients with discordant echocardiographic grading of aortic stenosis severity

|  | **Univariable** | | **Multivariable Model 1** | | **Multivariable Model 2** | |
| --- | --- | --- | --- | --- | --- | --- |
|  | **HR (95% CI)** | **P Value** | **HR (95% CI)** | **P Value** | **HR (95% CI)** | **P Value** |
| Age | 1.05 (1.02 to 1.08) | **<0.001** | 1.04 (1.02 to 1.07) | **<0.001** | 1.05 (1.02 to 1.08) | **0.001** |
| Sex | 1.07 (0.69 to 1.67) | 0.77 |  |  | 1.15 (0.  73 to 1.81) | 0.56 |
| Body-mass index | 1.02 (0.98 to 1.07) | 0.33 |  |  |  |  |
| Hypertension | 1.55 (0.94 to 2.66) | 0.10 |  |  |  |  |
| Smoking history | 1.04 (0.48 to 2.04) | 0.91 |  |  |  |  |
| Hyperlipidaemia | 1.71 (1.10 to 2.66) | **0.02** | 1.54 (0.98 to 2.41) | 0.59 | 0.61 (1.02 to 2.54) | **0.04** |
| Diabetes | 1.30 (0.80 to 2.06) | 0.28 |  |  |  |  |
| Chronic Kidney Disease | 1.86 (0.90 to 3.45) | 0.07 |  |  |  |  |
| Coronary Artery Disease | 1.37 (0.76 to 2.34) | 0.27 |  |  |  |  |
| Bicuspid Aortic Valve Disease | 0.56 (0.25 to 1.10) | 0.12 |  |  |  |  |
| Left Ventricular Ejection Fraction | 0.58 (0.31 to 1.22) | 0.12 |  |  |  |  |
| Peak aortic jet-velocity^a^ | 2.24 (0.75 to 7.14) | 0.16 |  |  | 2.67 (0.80 to 9.58) | 0.12 |
| High indexed calcium volume | 1.56 (1.00 to 2.40) | **0.04** | 1.58 (1.01 to 2.44) | **0.04** | 1.45 (0.91 to 2.30) | 0.11 |
| High calcium score | 1.48 (0.91 to 2.38) | 0.11 |  |  |  |  |
| High non-indexed calcium volume | 1.55 (0.97 to 2.42) | 0.06 |  |  |  |  |

^a^ log_2_ transformation was applied to achieve normal distribution. HR = hazard ratio.

**Supplemental Table 12**

Patient characteristics of the validation cohort

|  | | All | Men | Women | Non-severe | Severe |
| --- | --- | --- | --- | --- | --- | --- |
| Clinical | | 459 | 259 | 200 | 213 | 246 |
| Age | y | 74±10 | 74±10 | 75±10 | 72±11 | 76±9 |
| Male | % | 56 | 100 | 0 | 60 | 54 |
| Body-mass index | kg/m^2^ | 26±5 | 26±4 | 26±6 | 26±5 | 26±5 |
| Hypertension | % | 69 | 67 | 72 | 68 | 70 |
| Hyperlipidemia | % | 54 | 51 | 58 | 50 | 58 |
| Diabetes Mellitus | % | 30 | 31 | 29 | 29 | 31 |
| Current or ex-smoker | % | 27 | 38 | 9 | 33 | 24 |
| Coronary artery disease | % | 24 | 24 | 24 | 16 | 34 |
| Bicuspid aortic valve | % | 15 | 17 | 12 | 28 | 19 |
| Echocardiography | | | | | | |
| Peak velocity | m/s | 4.0 [2.8-4.5] | 4.0 [2.7-4.6] | 4.1 [2.9-4.5] | 2.8 [2.4-3.2] | 4.5 [4.2-4.9] |
| Mean gradient | mmHg | 37 [17-49] | 35 [16-49] | 39 [18-50] | 16 [12-23] | 48 [42-58] |
| Aortic valve area | cm^2^ | 0.90 [0.67-1.31] | 0.96 [0.68-1.33] | 0.85 [0.65-1.28] | 1.34 [1.16-1.63] | 0.69 [0.55-0.79] |
| Ejection fraction | % | 60 [56-65] | 60 [55-64] | 60 [56-65] | 62 [58-66] | 60 [55-63] |
| Computed Tomography | | | | | | |
| Calcium score* | AU | 1879 [941-3345] | 2264 [1150-4036] | 1630 [724-2824] | 858 [377-1395] | 3046 [1895-4467] |
| Indexed calcium volume | mm^3^/cm^2^ | 98 [32-201] | 115 [48-237] | 75 [20-166] | 32 [14-64] | 184 [110-302] |

* Calcium scores available in 305 patients.

**Supplemental Table 13**

Patient characteristics of the non-AVR and AVR groups

|  | | Non-AVR | AVR | p-value |
| --- | --- | --- | --- | --- |
| Clinical | | 680 | 468 |  |
| Age | y | 72±11 | 76±10 | <0.001 |
| Male | % | 63 | 46 | <0.001 |
| Body-mass index | kg/m^2^ | 26±5 | 26±5 | 0.456 |
| Hypertension | % | 67 | 74 | 0.022 |
| Hyperlipidemia | % | 39 | 69 | <0.001 |
| Diabetes Mellitus | % | 27 | 32 | 0.057 |
| Current or ex-smoker | % | 30 | 18 | 0.003 |
| Coronary artery disease | % | 17 | 46 | <0.001 |
| Bicuspid aortic valve | % | 16 | 20 | 0.125 |

AVR = aortic valve replacement
